# Evaluation of the anti-inflammatory effects of synthesised tanshinone I and isotanshinone I analogues in zebrafish

**DOI:** 10.1371/journal.pone.0240231

**Published:** 2020-10-06

**Authors:** Matthew J. Foulkes, Faith H. Tolliday, Katherine M. Henry, Stephen A. Renshaw, Simon Jones

**Affiliations:** 1 Department of Chemistry, The University of Sheffield, Sheffield, United Kingdom; 2 The Bateson Centre, The University of Sheffield, Sheffield, United Kingdom; 3 Department of Infection, Immunity & Cardiovascular Disease, The University of Sheffield, Sheffield, United Kingdom; Imperial College London, UNITED KINGDOM

## Abstract

During inflammation, dysregulated neutrophil behaviour can play a major role in a range of chronic inflammatory diseases, for many of which current treatments are generally ineffective. Recently, specific naturally occurring tanshinones have shown promising anti-inflammatory effects by targeting neutrophils *in vivo*, yet such tanshinones, and moreover, their isomeric isotanshinone counterparts, are still a largely underexplored class of compounds, both in terms of synthesis and biological effects. To explore the anti-inflammatory effects of isotanshinones, and the tanshinones more generally, a series of substituted tanshinone and isotanshinone analogues was synthesised, alongside other structurally similar molecules. Evaluation of these using a transgenic zebrafish model of neutrophilic inflammation revealed differential anti-inflammatory profiles *in vivo*, with a number of compounds exhibiting promising effects. Several compounds reduce initial neutrophil recruitment and/or promote resolution of neutrophilic inflammation, of which two also result in increased apoptosis of human neutrophils. In particular, the methoxy-substituted tanshinone **39** specifically accelerates resolution of inflammation without affecting the recruitment of neutrophils to inflammatory sites, making this a particularly attractive candidate for potential pro-resolution therapeutics, as well as a possible lead for future development of functionalised tanshinones as molecular tools and/or chemical probes. The structurally related β-lapachones promote neutrophil recruitment but do not affect resolution. We also observed notable differences in toxicity profiles between compound classes. Overall, we provide new insights into the *in vivo* anti-inflammatory activities of several novel tanshinones, isotanshinones, and structurally related compounds.

## Introduction

**Fig 1:**
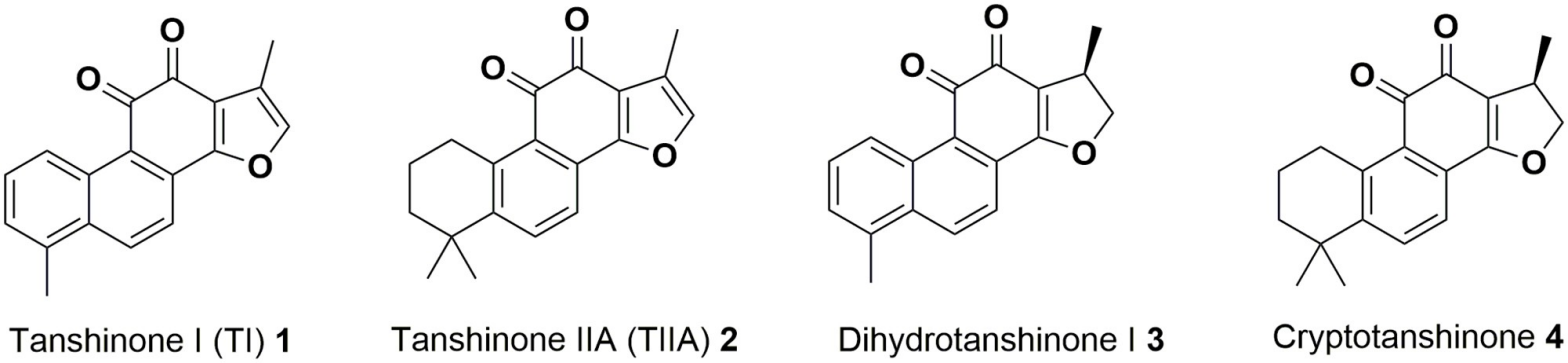
Structures of some naturally occurring tanshinones 1–4.

Tanshinones are a group of aromatic diterpenoid compounds comprising four fused rings; two of these form a naphthalene or tetrahydronaphthalene moiety, the third ring usually bears an *ortho*- (or *para*-) quinone, and the fourth ring is a furan or dihydrofuran. Tanshinone I (TI) **1** is one of the main known tanshinones found naturally in the plant *Salvia miltiorrhiza* (also known as Chinese red sage or danshen), whilst others include tanshinone IIA (TIIA) **2**, and the dihydro counterparts, dihydrotanshinone I **3** and cryptotanshinone **4** (Fig 1). The plant has been traditionally used as a remedy in Chinese herbal medicine since as early as the first century AD, mainly for treatment of cardiovascular disease [1]. Isolation of milligram quantities of tanshinones from the plant usually requires serial extractions from the dried roots, followed by chromatographic separation [2–5].

Isolated tanshinones have been reported to exert various biological activities both *in vitro* and *in vivo*. In particular, for TI **1** these include anti-inflammatory effects [6–9], antimicrobial activity [10, 11], cytotoxicity towards cancer cells [12–15], neuroprotective effects [16], benefits for learning and memory enhancement [17], antioxidant activity [18, 19], and the ability to induce cell apoptosis [12, 20, 21]. In most cases however, the mechanism of action is poorly understood, and there is often limited or no knowledge of structure-activity relationships (SARs).

**Fig 2:**
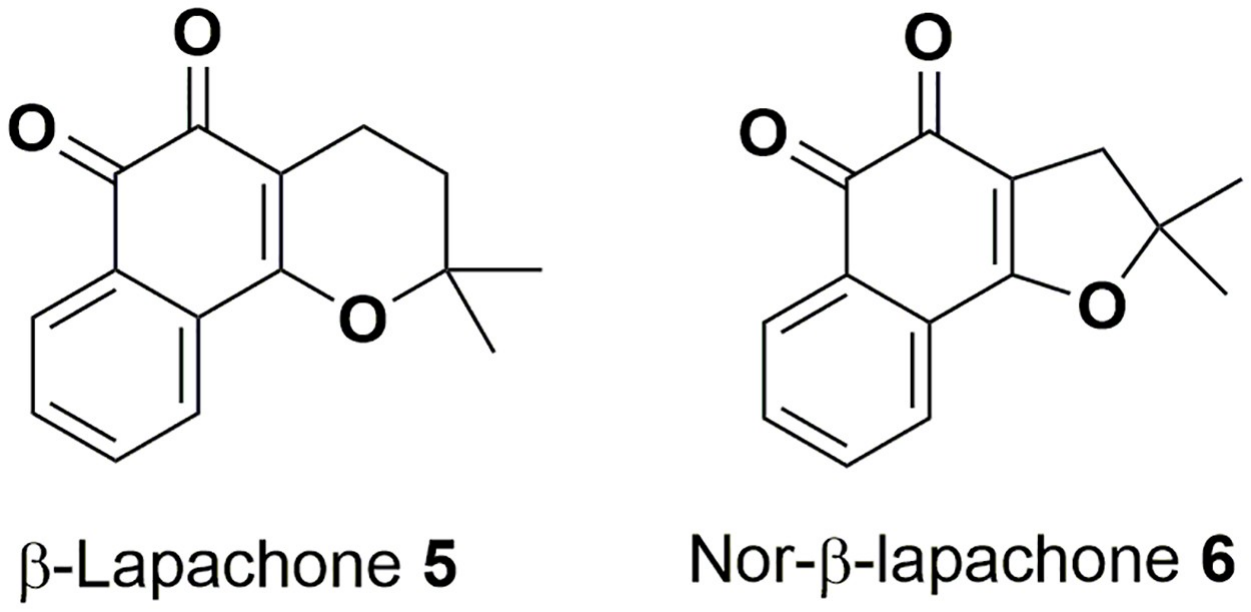
Structures of β-lapachone 5 and nor-β-lapachone 6.

β-Lapachone **5** and nor-β-lapachone **6** are molecules with a high degree of structural similarity to tanshinones (Fig 2). β-Lapachone **5** is a naturally-occurring *ortho*-quinone compound obtained from the bark of the lapacho tree *Tabebuia avellanedae* in Brazil, a plant used for centuries as a traditional medicine for various treatments, including as an analgesic, an anti-inflammatory agent, an antineoplastic agent, and a diuretic [22, 23]. In more recent studies, β-lapachone **5** has exhibited anti-inflammatory effects [24–29], as well as various anti-cancer activities [30–33], anti-angiogenesis activity [34, 35], antimicrobial effects [36–38], and wound healing properties [39]. In addition, both β-lapachone **5** and TIIA **2** have been found to act as inhibitors of NAD(P)H quinone oxidoreductase 1 (NQO1), leading to an increase in cellular NAD(+) [40–42]. This recent finding connecting the two structures, alongside the clear structural similarities, may suggest that these two classes of compounds could exhibit common biological activities *in vivo*. In contrast, reports of previous studies involving nor-β-lapachone **6** are scarce. Although nor-β-lapachone **6** is considered an anti-cancer drug candidate [43, 44], at this time there appear to be no studies involving nor-β-lapachone **6** in relation to inflammation, neutrophils or zebrafish, perhaps because β-lapachone **5** is a naturally occurring compound, whereas nor-β-lapachone **6** is not.

Neutrophils, the most abundant white blood cell in humans, play a vital role in the inflammatory response. Their recruitment to the site of pathogen invasion or tissue injury is essential for elimination of invading pathogens by various mechanisms [45–49]. Strict regulation of neutrophil function is required: whilst these processes are required for effective host defence and response, they also need to be limited to avoid persistent inflammation [50]. Subsequent timely neutrophil clearance from this site, as part of the resolution of inflammation, is also necessary for healthy recovery [51, 52]. Failure of this process can result in retention of persistent inflammatory neutrophils, which can have damaging effects on the body, playing a key role in increasingly prevalent inflammatory diseases, for example in chronic obstructive pulmonary disease (COPD) [53, 54], asthma [55, 56], or rheumatoid arthritis [57, 58]. Current treatments for unresolved neutrophilic inflammation are non-specific, poorly effective, and can exhibit many undesired side-effects [59, 60]; thus there is an unmet need for new neutrophil-specific clinical treatments.

The zebrafish (*Danio rerio*) is an excellent *in vivo* model for the study of inflammation and neutrophil biology [61], and has been utilised effectively in a number of recent studies in the discovery of previously unidentified compounds with anti-inflammatory properties *in vivo* [62–65]. Screening in a transgenic zebrafish inflammation model identified the naturally occurring TIIA **2** as a compound which significantly accelerated resolution of neutrophilic inflammation *in vivo*, without affecting initial neutrophil recruitment or total neutrophil number [63]. The related molecule cryptotanshinone **4** displayed broadly similar anti-inflammatory effects, although this compound also decreased initial neutrophil recruitment. However, no other tanshinones have been investigated in this manner, meaning that no knowledge of any analogous effects of tanshinones based on TI **1** currently exists in the literature; this is also true for the isotanshinones.

Naturally occurring tanshinones are also inherently carbon-rich and planar, meaning they possess sub-optimal solubilities and physicochemical properties. To this end, incorporation of an additional heteroatom and a possible functional group handle for development of related chemical probes and/or molecular tool compounds, provides further rationale for development and evaluation of non-natural tanshinone and isotanshinone analogues. As little is understood about the *in vivo* anti-inflammatory effects of these compounds, besides the naturally occurring, unfunctionalised tanshinones, an increased understanding of any SARs would provide increased drug discovery opportunities for future research. Furthermore, SARs both within and between different tanshinone classes (TI and TIIA) in this model are currently unexplored. We here report synthesis of a set of tanshinone I and isotanshinone I analogues, and use of a zebrafish model for evaluation of the effects of such compounds on different stages of the *in vivo* inflammatory response utilising a phenotypic screening approach, as well as initial assessment of any *in vivo* toxicity.

## Results and discussion

### Chemical synthesis of TI analogues and isomers

**Fig 3:**
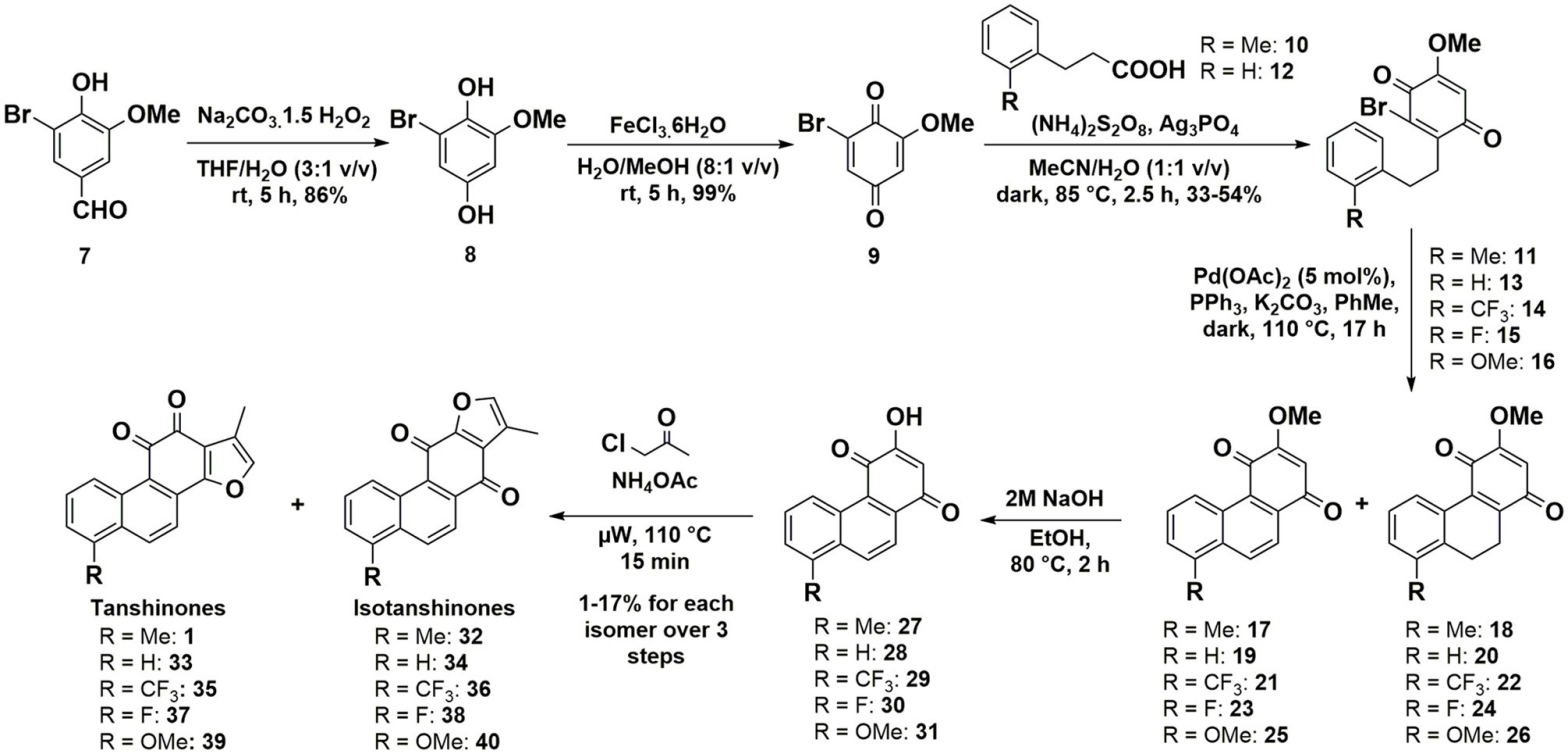
Synthesis of tanshinones and isotanshinones.

Synthesis of TI analogues utilised a route similar to that of Jiao and co-workers [66], but with important modifications (Fig 3). Commercially available 5-bromovanillin **7** was first converted to the hydroquinone **8** *via* Baeyer-Villiger oxidation and subsequent ester hydrolysis, followed by Fe(III)-mediated oxidation to the corresponding benzoquinone **9** [67]. Both transformations were performed on a multi-gram scale (up to 25 grams) in high yield, with a lack of chromatographic purification representing a more time- and cost-effective large-scale isolation of the desired benzoquinone **9** compared to previous reports. This material was treated with various substituted carboxylic acids in a radical decarboxylative coupling. Initial use of 3-(2-methylphenyl)propionic acid **10**, with conditions identified from the literature [66], resulted in low yields of alkylation product **11** of approximately 20% after chromatographic purification. Reaction optimisation, through systematic variation of numerous reaction parameters using the unsubstituted 3-phenylpropionic acid **12**, in a Design of Experiments (DoE) approach (further information provided in the S1 File), led to improved yields of around 40%, including a 42% yield of alkylation product **11**. Throughout these studies, although formation of a small quantity (< 10%) of doubly-alkylated product was observed, none of the alternative mono-alkylated product (alkylated adjacent to the methoxide group) was seen, which was consistent with previous reports, and likely a directing effect due to the bromide substituent [66, 68].

Use of different carboxylic acids in this step was then considered. The choice of several different substituted carboxylic acids, which would eventually provide various final tanshinone and isotanshinone molecules, was based on several factors. One of these was a desire to include a mixture of electron-withdrawing, electron-donating, and electron-neutral groups in the final molecules, thereby perhaps changing the properties of the tanshinones as a whole. The inclusion of fluorine-containing groups was also of interest, as it was considered that this may have some effect on *in vivo* metabolism, in comparison to the solely hydrogen-containing analogues, although detailed studies and predictions of this nature were beyond the scope of this work. Variation of the substituent on the phenyl ring of the carboxylic acid in this step allowed for modification of a position in the final tanshinone as far away as possible from the most chemically and biologically interesting part of the molecule, the *ortho*-quinone moiety. It was hypothesised that modifying in such a position would thereby have a minimal effect on the interaction of such compounds with their molecular target(s) *in vivo*. Furthermore, carboxylic acid choice was limited to an extent by the commercial availability of such substrates.

Thus, the optimised conditions were applied to the reaction for other substituted carboxylic acids, and products bearing methyl **11**, hydrogen **13**, trifluoromethyl **14** and fluoro **15** substituents were all formed in consistently moderate yields of 38–54%. For the methoxy substituent, the alkylation product **16** was formed in an inseparable 60:40 mixture with the starting material **9**, corresponding to an approximate 33% overall yield of desired product **16** (see S1 File for further details).

Employing the conditions for the intramolecular Heck reaction used in the literature for the methyl-substituted bromide **11** [66], only gave the desired cyclisation product in a maximum of 40% yield after purification, as a mixture of aromatised **17** and non-aromatised **18** compounds. This was unacceptably low given the high quantities of palladium catalyst used (45 mol%) and use of a much smaller quantity of catalyst was desired. Optimisation was carried out using the unsubstituted variant **13** as a model system (see S1 File for further details); whilst changing the phosphine ligand or adding a co-oxidant to the mixture had no beneficial effect on the yield, performing the reaction at a much lower concentration (approximately 0.01 M), led to a greatly improved of 74% when just 10 mol% of catalyst was used, and this was conserved (73% yield) when the catalyst loading was reduced to 5 mol%. These results thus suggested that this particular intramolecular reaction was especially sensitive to concentration effects. In addition, throughout these studies, varying ratios of aromatised **19** and non-aromatised products **20** were obtained; however, any non-aromatised product **20** was converted to the fully aromatised product in subsequent steps.

The improved Heck reaction conditions were used to prepare the corresponding analogues **17–26**. Whilst mass returns were good for the methyl- and hydrogen-substituted variants **17–18** and **19–20**, heteroatom-substituted compounds were lower. For the methoxy-substituted compound **16**, residual benzoquinone **9** was able to be removed, and the desired products **25–26** were successfully produced although also with a low mass return (see S1 File for further details).

Demethylation of the mixture of inseparable methyl ethers **17–18** was achieved by heating at reflux in a 2 M sodium hydroxide solution with ethanol, with concomitant aromatisation successfully yielding solely the desired alcohol **27**. This avoided the need for additional oxygen gas to be supplied to the reaction, as previously utilised in the literature [66], and worked well for all analogues **27–30**, and for the methoxy compound **31**, where only the single desired demethylation product was observed.

In a change of conditions from the literature procedure, reaction of the alcohol **27** with chloroacetone as the solvent with 1 equivalent of ammonium acetate resulted in isolation of the desired TI **1**, together with the isomeric isotanshinone I (iso-TI) **32** [66, 69–72]. The two isomers were each isolated in 12% yield over three steps, presumably formed *via* tautomeric intermediates. This reaction worked consistently for each of the different analogues to yield tanshinones **1, 33, 35, 37, 39** and isotanshinones **32, 34, 36, 38, 40**. Yields were higher for the electron-neutral methyl- and hydrogen-substituted compounds, and were somewhat lower for the heteroatom-substituted variants. Only a few examples of isotanshinones are known in the literature, including iso-TI **32** and isotanshinone IIA **41** [69, 70, 72, 73], and related isotanshinones [74, 75]. These all occur naturally in *Salvia miltiorrhiza*, although isotanshinone I **32** and isotanshinone IIA **41** have been produced synthetically [69, 70]. However as a class of compounds, isotanshinones are very much underexplored, both in terms of synthesis and biological studies. Thus, this synthesis provided efficient access to a range of both substituted tanshinone I analogues and isotanshinones of interest, most of which are completely novel compounds.

**Fig 4:**
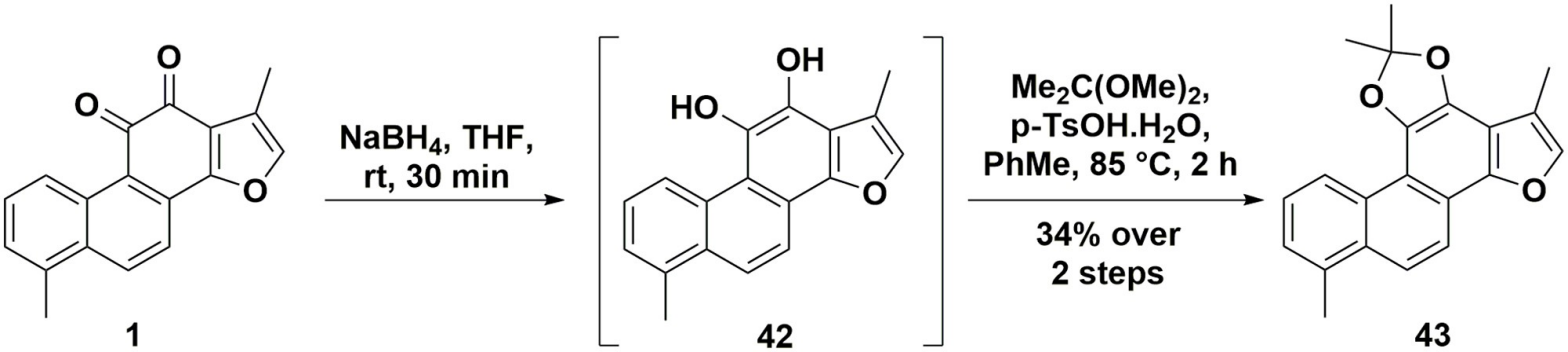
Synthesis of the TI-acetal 43.

To investigate the importance of the *ortho*-quinone moiety of TI **1** for its biological activity (as a functional group common to all major tanshinones), a ‘masked’ analogue lacking this functionality was desired. Synthesis of a dimethyl acetal analogue of TI **1** was readily accomplished in a two-step procedure (Fig 4) by reduction of the *ortho*-quinone **1** with sodium borohydride, followed by quickly treating the diol **42** with 2,2-dimethoxypropane and acid giving the stable acetal **43** in 34% yield over two steps.

### Chemical synthesis of lapachols and β-lapachones

**Fig 5:**
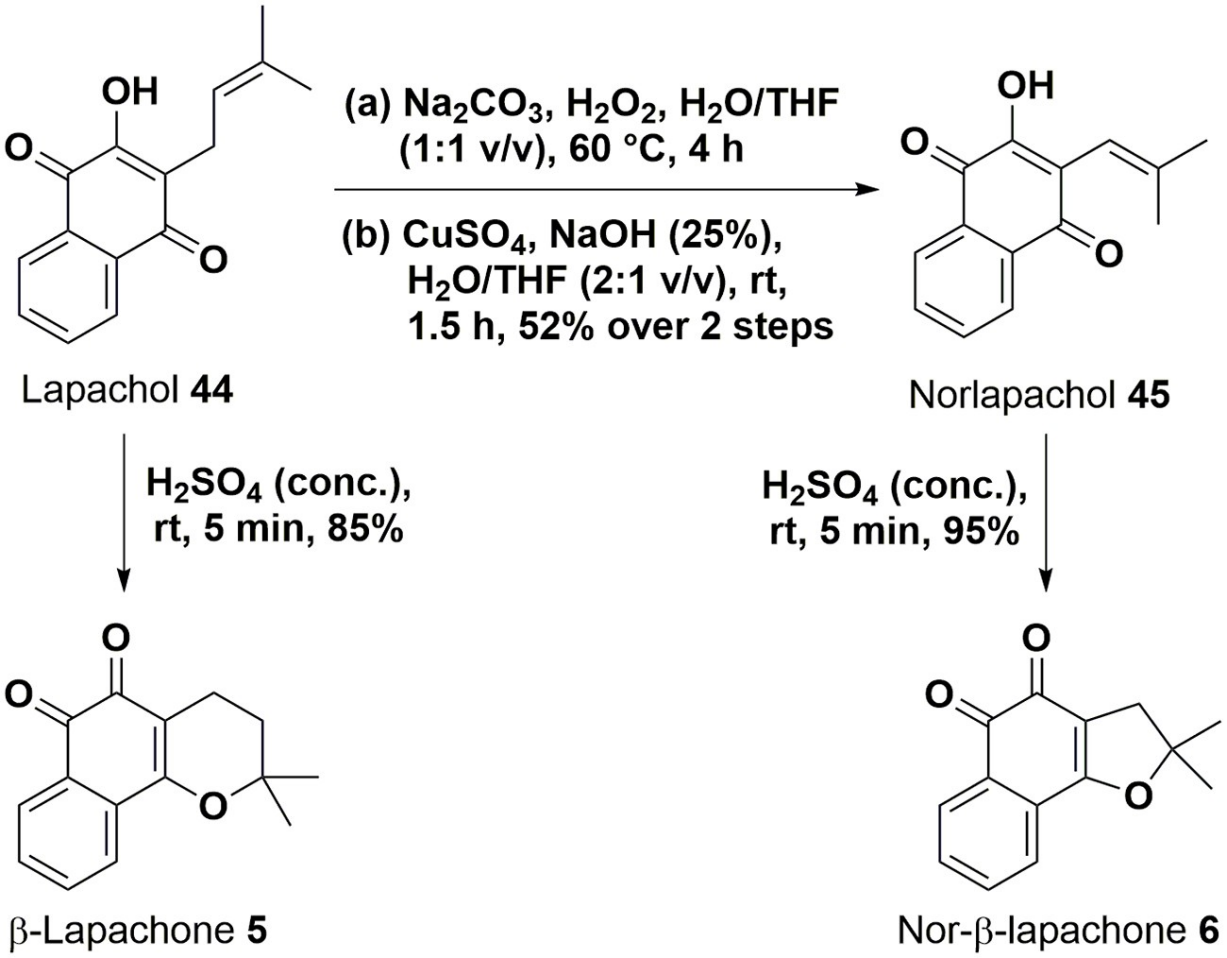
Synthesis of norlapachol 45 and β-lapachones 5, 6.

Structurally related β-lapachones were synthesised readily from commercially sourced lapachol **44** (Fig 5). β-Lapachone **5** was synthesised in a single step and in high yield from treatment of lapachol **44** with concentrated sulfuric acid.

Norlapachol **45** was also produced from lapachol, *via* a Hooker oxidation, in an unoptimized yield of 52% after recrystallisation. Finally, nor-β-lapachone **6** was synthesised from its corresponding precursor norlapachol **45**, again *via* acid-mediated cyclisation, in a near-quantitative yield of 95% after simple purification by trituration.

### Evaluation of the *in vivo* anti-inflammatory effects of TI and TIIA

**Fig 6:**
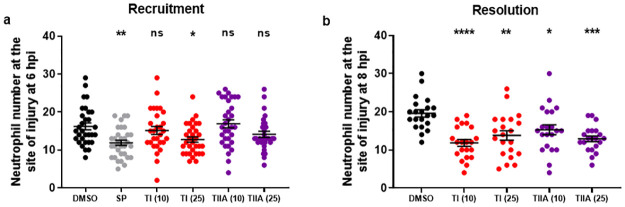
Effects of TI 1 and TIIA 2 on a) initial neutrophil recruitment and b) resolution of neutrophilic inflammation in a zebrafish inflammation model. For **a**), tailfin transection was performed on transgenic zebrafish larvae, *TgBAC(mpx*:*GFP)i114* (3 days post-fertilisation, dpf), which were subjected to compound treatments immediately: DMSO (0.5%), SP600125 (30 μM) and TI **1** and TIIA **2** (10, 25 μM as indicated in brackets). Neutrophil number at the site of injury was assessed at 6 hpi, by counting GFP+ cells. For **b**), tailfin transection was performed on zebrafish larvae (3 dpf), and at 4 hpi, good responders were subjected to compound treatments: DMSO (0.5%), TI **1** and TIIA **2** (10 μM, 25 μM as indicated in brackets). Neutrophil number at the site of injury was assessed at 8 hpi by counting GFP+ cells. For both graphs, data shown as mean ± SEM; *n* = 21–32 larvae per group from 3–4 independent experiments per graph. * *P* < 0.05, ** *P* < 0.01, *** *P* < 0.001, **** *P* < 0.0001, compared to the DMSO vehicle control (one-way ANOVA with Dunnett’s multiple comparison post-test).

To explore the anti-inflammatory effects of the different tanshinone sub-classes, TI **1** and TIIA **2** were first evaluated in a transgenic zebrafish model of inflammation (Fig 6, Table 1), as previously described [63, 64]. For neutrophil recruitment experiments, the commercially available compound SP600125 (SP) was used as the positive control, as a selective and cell-permeable inhibitor of c-Jun N- terminal kinase (JNK), which prevents activation of various inflammatory genes [76, 77]. Investigation of the effects of the tanshinones on initial recruitment of neutrophils to the site of tailfin injury (Fig 6a) revealed that TI **1** (25 μM) reduced the number of neutrophils recruited at 6 hours post injury (hpi), although had no effect at 10 μM. TIIA **2**, however, did not significantly affect neutrophil recruitment at either 10 or 25 μM, in agreement with previous findings for TIIA **2** using this model [63]. For experiments investigating resolution of neutrophilic inflammation *in vivo* (Fig 6b), TIIA **2** (25 μM) was used as the positive control compound, as this has previously been shown to exhibit highly significant pro-resolution activity in this zebrafish model of inflammation [63]. Both TI **1** and TIIA **2** accelerated resolution of neutrophilic inflammation, at 10 and 25 μM.

**Table 1:** Summary of the effects of various tanshinones and isotanshinones on neutrophil recruitment and resolution of neutrophilic inflammation *in vivo*.

Compound	Effect on recruitment	Effect on resolution
TI **1**	Reduced	Enhanced
TIIA **2**	No effect	Enhanced
Iso-TI **32**	Reduced	Enhanced
6-hydro-TI **33**	No effect	No effect
6-trifluoromethyl-TI **35**	No effect	No effect
6-fluoro-TI **37**	No effect	No effect
6-methoxy-TI **39**	No effect	Enhanced
4-hydro-iso-TI **34**	No effect	No effect
4-trifluoromethyl-iso-TI **36**	Reduced	No effect
4-fluoro-iso-TI **38**	Reduced	No effect
4-methoxy-iso-TI **40**	No effect	No effect
TI-acetal **43**	No effect	Enhanced

### Evaluation of the effects of TI analogues and isomers on neutrophil recruitment and resolution *in vivo*

**Fig 7:**
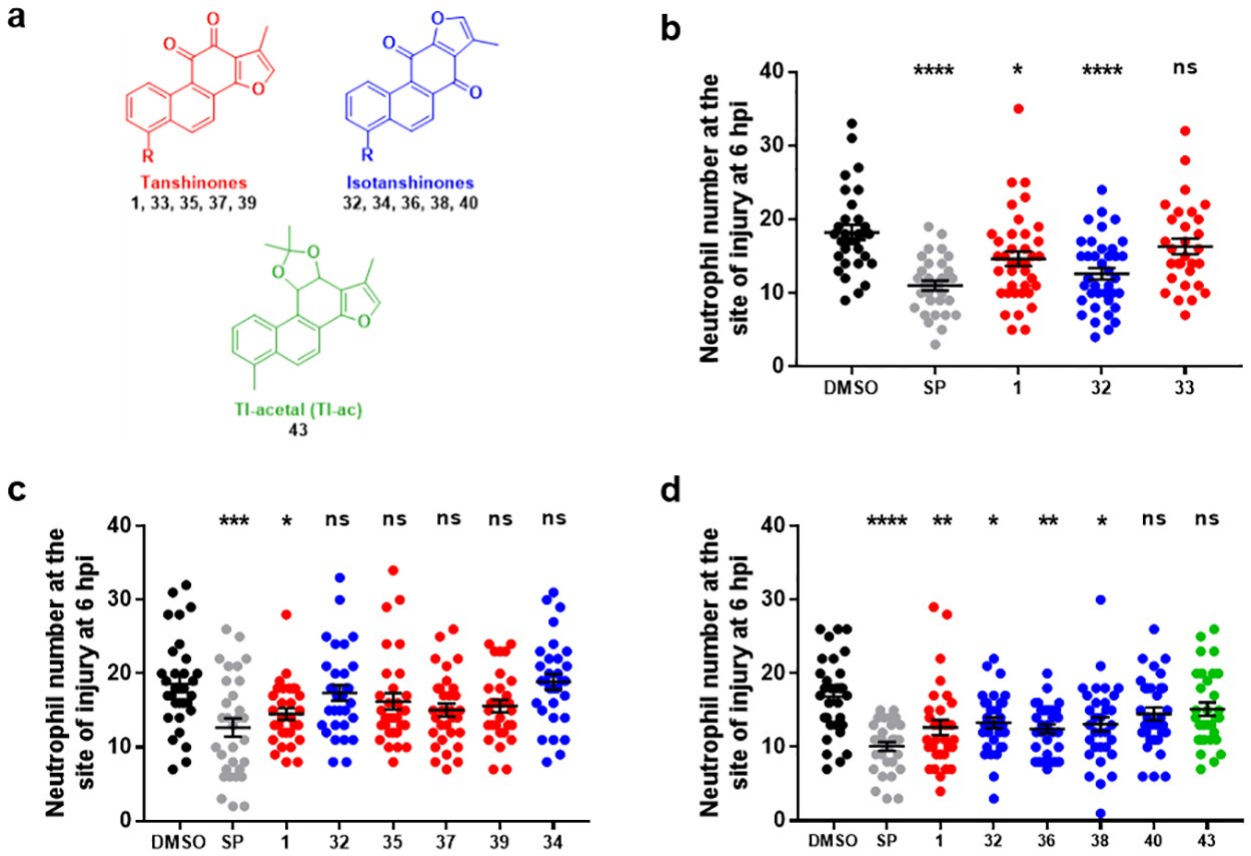
Effects of TI analogues and isomers on neutrophil recruitment. **a**) Structures of evaluated tanshinones (red), isotanshinones (blue) and the TI-acetal (green). **b**)-**d**) Tailfin transection was performed on transgenic zebrafish larvae, *TgBAC(mpx*:*GFP)i114* (3 dpf) which were subjected to compound treatments immediately: DMSO (0.5%), SP600125 (30 μM) and compounds **1**, **32–40**, **43** (25 μM). Neutrophil number at the site of injury was assessed at 6 hpi by counting GFP+ cells. Data shown as mean ± SEM; *n* = 30–39 larvae from 4 independent experiments per graph. * *P* < 0.05, ** *P* < 0.01, *** *P* < 0.001, **** *P* < 0.0001, ns not significant, in comparison to the DMSO vehicle control (one-way ANOVA with Dunnett’s multiple comparison post-test).

The synthesised TI analogues and isomers **1**, **32**–**40**, **43** were evaluated for any effects on neutrophil recruitment in the same zebrafish inflammation model (Table 1, Fig 7), tested using the same experimental conditions across three sets due to practical constraints on experiment size, and again with DMSO and SP as the negative and positive control treatments, respectively. As expected, TI **1** significantly reduced the number of neutrophils recruited to the site of injury in all three datasets (Fig 7b–7d). Generally, treatment with iso-TI **32** also resulted in a significant decrease in the number of neutrophils recruited (Fig 7b–7d), with one exception.

The tanshinone analogues **33, 35, 37, 39** are derived from the parent TI structure **1** (Table 1, Fig 7b and 7c, data shown in red). Hydrogen substitution (compound **33**) did not change neutrophil recruitment compared with the DMSO control. Similarly, compounds substituted with trifluoromethyl (**35**), fluorine (**37**) or methoxy (**39**) groups had no effect on neutrophil recruitment.

The isotanshinones **34, 36, 38, 40** are derived from the parent iso-TI structure **32** (Table 1, Fig 7c and 7d, relevant data depicted in blue). Replacement of the 4-methyl group with a hydrogen atom (**34**) again resulted in the loss of any effect on neutrophil recruitment to the site of injury. However, replacement of this methyl group with either a trifluoromethyl group (**36**) or a fluorine atom (**38**) resulted in significantly fewer recruited neutrophils in each case, although the methoxy- substituted analogue **40** had no significant effect on recruitment. The TI-acetal **43** also had no significant effect on neutrophil recruitment to the site of injury (Fig 7d), possibly due to a lack of the active form TI **1** *in vivo*.

In general (except for iso-TI **32** in a single dataset), compounds with a methyl substituent have a clear effect in reducing neutrophil recruitment to the site of injury, yet removal of any functionality at this position leads to a complete loss of biological effect, possibly due to decreased aqueous solubility, and/or loss of a particular interaction with the molecular target(s) *in vivo*. Compounds with different substituents in the modified position show variable effects on neutrophil recruitment *in vivo*, with some isotanshinones resulting in decreased recruitment, unlike the corresponding tanshinones.

**Fig 8:**
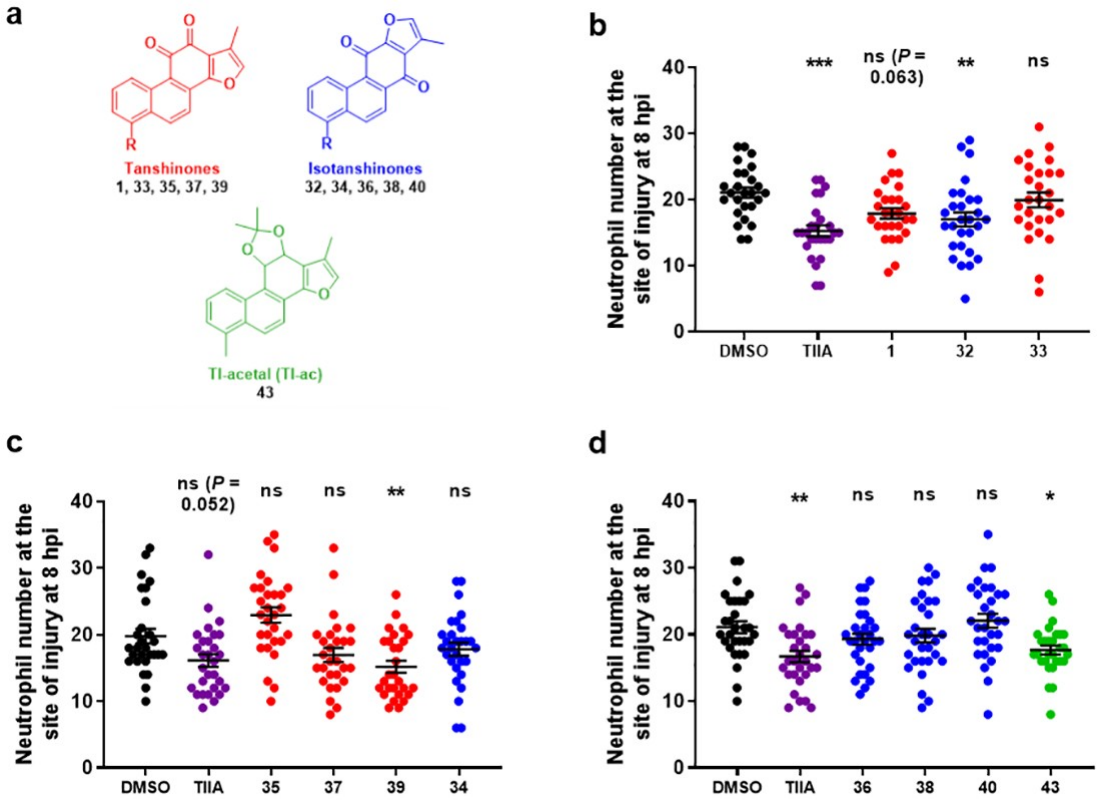
Effects of TI analogues and isomers on resolution of neutrophilic inflammation. **a**) Structures of tanshinones (red), isotanshinones (blue) and TI-acetal (green). **b**)-**d**) Tailfin transection was performed on transgenic zebrafish larvae, *TgBAC(mpx*:*GFP)i114* (3 dpf), and at 4 hpi, good responders were subjected to compound treatments: DMSO (0.5%), TIIA **2** (25 μM) and compounds **1**, **32–40**, **43** (25 μM). Neutrophil number at the site of injury was assessed at 8 hpi by counting GFP+ cells. Data shown as mean ± SEM; *n* = 26–30 larvae per group from 4 independent experiments per graph. * *P* < 0.05, ** *P* < 0.01, *** *P* < 0.001, ns not significant, in comparison to the DMSO control (one-way ANOVA with Dunnett’s multiple comparison post-test).

The synthesised TI analogues and isomers **1, 32–40, 43** were then evaluated in a similar zebrafish model of inflammation resolution (Table 1, Fig 8). For resolution experiments, TIIA **2** was again used as the positive control compound (Fig 8b–8d). Firstly considering the *ortho*-quinone tanshinone compounds **1, 33, 35, 37, 39** (Fig 8b and 8c, data shown in red), in larvae treated with TI **1**, there was a trend towards lower neutrophil numbers at the site of injury, compared to the DMSO-treated larvae (*P* = 0.063). Replacement of the methyl group in the 6-position (**1**) with a hydrogen atom (**33**) resulted in no effect on resolution *in vivo*. Related compounds containing a trifluoromethyl group (**35**) or fluorine atom (**37**) also showed no effect. Interestingly, for the 6-methoxy substituted tanshinone **39**, there was a clear reduction in neutrophil number at the site of injury at 8 hpi, in comparison to the DMSO control-treated larvae, indicating that this compound accelerates resolution of inflammation. This compound **39** was of particular interest as it promoted resolution of neutrophilic inflammation but did not affect initial neutrophil recruitment, making this compound a more promising candidate for further investigation of specific pro-resolution treatments which do not impair host-defence. Furthermore, the methoxy group also provides a possible functionalisation point, which could be useful for developing tool compounds and molecular probes. The methoxy group also likely confers improved aqueous solubility to the molecule.

For the isotanshinones **32, 34, 36, 38, 40**, (Fig 8b–8d, data shown in blue), iso-TI **32** significantly accelerated neutrophilic inflammation resolution, whilst the unsubstituted compound **34** again had no effect. The remaining isotanshinones **36, 38, 40** had no effect on inflammation resolution, irrespective of the functional group in the 4-position. Treatment with the TI-acetal **43** resulted in a reduction in neutrophil number at the site of injury (Fig 8d). One possible explanation is that the acetal **43** was (partially) hydrolysed and metabolised back to the parent TI **1** *in vivo*, which then exerted a pro-resolution effect as seen previously (Fig 6b). These results suggest that a substituent in the 6- or 4- position for tanshinones and isotanshinones respectively, is required for activity, the nature of which is particularly important for pro-resolution activity.

Overall, both substituted tanshinones and isotanshinones exhibit differential effects on recruitment and resolution (Table 1). This is not particularly surprising, as molecules which affect neutrophil recruitment likely interact with a different molecular target to those which affect resolution of neutrophilic inflammation. Of particular note are: the abilities of both of the parent compounds TI **1** and iso-TI **32** to decrease neutrophil recruitment to the site of injury and accelerate inflammation resolution; isotanshinones **36** and **38** both resulting in decreased neutrophil recruitment to the injury site yet having no effect on resolution of neutrophilic inflammation; and the methoxy-substituted tanshinone **39** exhibiting no effect on neutrophil recruitment, yet significantly promoting inflammation resolution (analogous to the findings observed for TIIA **2**). This suggests that this particular compound may be an interesting candidate to focus on to investigate compounds which specifically target inflammation resolution, as well as providing a lead for synthesis of future functionalised tanshinones. In addition, the methoxy group of this compound may be improving the aqueous solubility of this molecule, compared to previously studied naturally occurring tanshinones.

Comparison of the fluorine-containing compounds with their non-fluorine-containing analogues could, on some level, provide insights into how *in vivo* metabolism may affect biological activity. Thus, whereas TI **1** affected both neutrophil recruitment and resolution, neither the unsubstituted tanshinone **33**, the trifluoromethyl-substituted variant **35**, nor the fluorine-substituted compound **37** significantly affected either process. This may suggest that the methyl group is important for biological for activity, since its removal or replacement with fluorine atoms (in either the methyl group or instead of an aryl hydrogen) both resulted in loss of *in vivo* activity. Meanwhile, for the isotanshinones, replacement of the methyl group with either a trifluoromethyl group (compound **36**) or a fluorine atom (compound **38**) did not affect neutrophil recruitment, with both compounds resulting in reduced neutrophil recruitment, like the parent iso-TI **32**. However, whereas inflammation resolution was enhanced for iso-TI 32, both of the fluoro-containing compounds **36** and **38** had no effect on resolution. This suggests that for isotanshinones, changing the methyl group to a fluoro-containing group can be tolerated in relation to effects on neutrophil recruitment, but not resolution. This may also suggest in this case that *in vivo* C-H bond metabolism (or some other factor relating to C-F bond substitution) is important for acceleration of inflammation resolution, but not for neutrophil recruitment. Yet, with the tanshinones, the results may suggest that C-H bond metabolism (or other factor(s)) may be important for effects on both neutrophil recruitment and resolution. Clearly, further work would be required to explore this in more detail.

Besides efficacy, another important consideration in drug discovery and development is that of toxicity; indeed, these are the two main reasons accounting for the vast majority of the high drug attrition rates in the pharmaceutical industry [78–80]. One of the great advantages of using this *in vivo* zebrafish model to evaluate potential anti-inflammatory compounds is that it also allows an early stage qualitative assessment of any side-effects on a living organism. Accordingly, the general health of treated zebrafish larvae was also observed for all experiments. At aqueous concentrations of 25 μM, none of the evaluated tanshinones and isotanshinones resulted in any larval toxicity; larvae were generally healthy in all treatments, as determined by visual inspection of general body shape, tail shape, presence of circulation, and heartbeat. However, it is important to note that toxicity was only assessed at the end of the experiment, after 4–6 hours immersion in the compound of interest. A thorough toxicity experiment would require leaving the fish in the compound solution for longer periods.

### Evaluation of the effects of lapachols and β-lapachones on neutrophil recruitment and resolution *in vivo*

**Fig 9:**
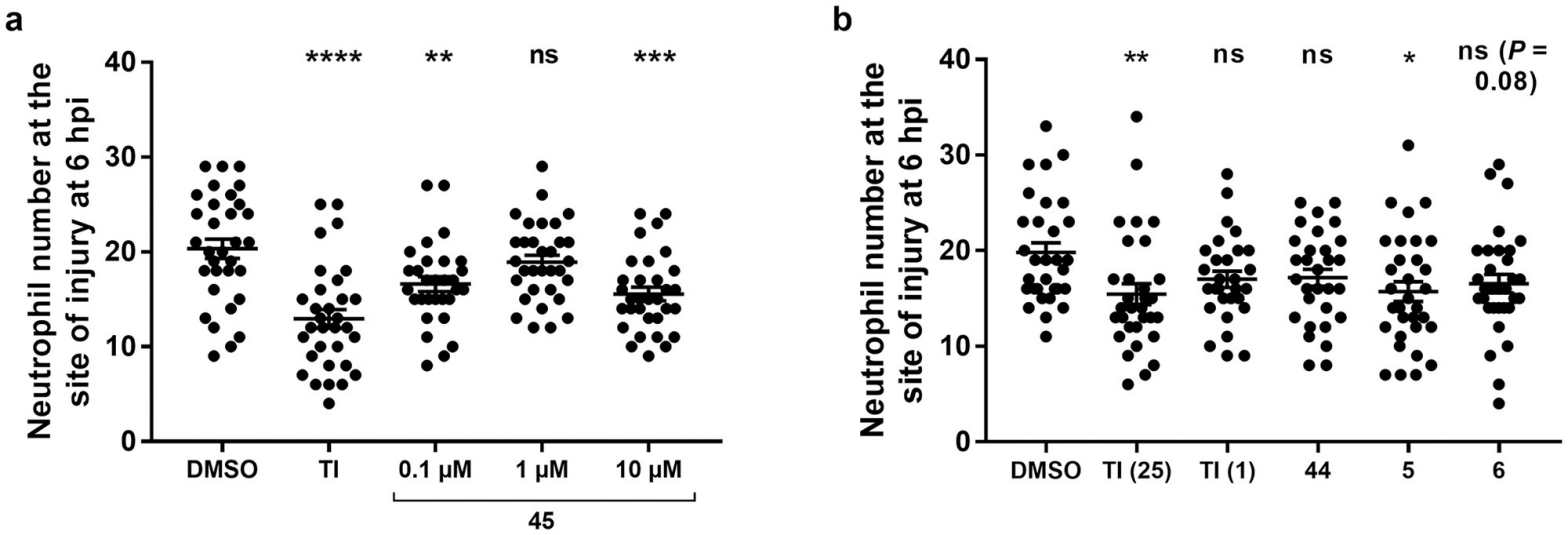
Effects of a) norlapachol 45 and b) lapachol 44, β-lapachone 5 and nor-β-lapachone 6 on neutrophil recruitment. Tailfin transection was performed on transgenic zebrafish larvae, *TgBAC(mpx*:*GFP)i114* (3 dpf) which were subjected to compound treatments immediately: DMSO (0.5%), TI **1** (25 μM; also 1 μM for **b** only, as indicated in brackets), norlapachol **45** (**a**; 0.1, 1, 10 μM as indicated) and lapachol **44**, β-lapachone **5** and nor-β-lapachone **6** (**b**; 1 μM). Neutrophil number at the site of injury was assessed at 6 hpi by counting GFP+ cells. Data shown as mean ± SEM; *n* = 29–32 larvae from 4 independent experiments per graph. * *P* < 0.05, ** *P* < 0.01, *** *P* < 0.001, **** *P* < 0.0001, ns not significant, in comparison to the DMSO control (one-way ANOVA with Dunnett’s multiple comparison post-test).

Norlapachol **45**, lapachol **44**, β-lapachone **5**, and nor-β-lapachone **6** were also evaluated in neutrophil recruitment experiments (Table 2, Fig 9). To explore possible dose-response effects, norlapachol **45** was tested at 0.1, 1, and 10 μM; a higher dose of 25 μM appeared to result in larval abnormalities. Similarly, the remaining three compounds were all evaluated at 1 μM. This stood in contrast to the use of the various tanshinones at 25 μM previously, in which larvae were all healthy at this concentration, and is a worthwhile consideration when determining the viability of such molecules further in the drug discovery and development process. These observations suggest that the structural differences between these compounds have important consequences on their toxicity profiles *in vivo*. This could be due to differences in molecular target interactions, differences in larval penetration, and/or differences in neutrophil penetration, possibly due to interactions with relevant drug transporter proteins [81]. TI **1** (25 μM) was used as the positive control, based on the previous findings and to provide a synthetic control compound. Norlapachol **45** reduced the number of neutrophils recruited to the site of injury at both 10 μM and 0.1 μM (Fig 9a). Given the structural differences between norlapachol **45** and tanshinones, norlapachol **45** may have worked in a different way to tanshinones such as TI **1**, possibly by acting on a different protein target. Recruitment was not significantly reduced at 1 μM. The difference between the effects observed at 1 μM and 0.1 μM may reflect the natural variability in this type of *in vivo* experiment, despite all efforts to control for this as far as reasonably practicable.

**Table 2:** Summary of the effects of norlapachol 45, lapachol 44, β-lapachone 5, and nor-β-lapachone 6 on neutrophil recruitment and resolution of inflammation *in vivo*.

Compound	Concentration / μM	Effect on recruitment	Effect on resolution
Norlapachol **45**	25	*Toxic*	Enhanced (***)
	10	Reduced (***)	Enhanced (*)
	1	No effect	No effect
	0.1	Reduced (**)	No effect
Lapachol **44**	1	No effect	No effect
β-Lapachone **5**	1	Reduced (*)	No effect
Nor-β-Lapachone **6**	1	No effect	No effect

The remaining three compounds were evaluated in the same way but at 1 μM (Fig 9b). TI **1** was used at both 25 μM (as a known positive control) and 1 μM, to allow comparison of the effects of this compound to the other compounds tested at the same dosage. β-Lapachone **5** led to a significant reduction in the number of neutrophils recruited to the site of injury. However, neither nor-β-lapachone **6** nor lapachol **44** reduced neutrophil recruitment, nor did TI **1** at this concentration. No signs of toxicity were observed amongst the larvae of any of the treatment groups.

These results were particularly interesting, as β-lapachone **5** is an *ortho*-quinone compound which is structurally similar to TI **1**, and led to reduced neutrophil numbers at the injury site, as observed with TI **1** at a 25 μM concentration. However, TI **1** showed no effect when used at a concentration of 1 μM, suggesting that β-lapachone **5** may have been more active than TI **1** *in vivo*. This would clearly require further investigation, but is of interest given the toxicity issues observed with the β-lapachones yet not with TI **1** in this study.

**Fig 10:**
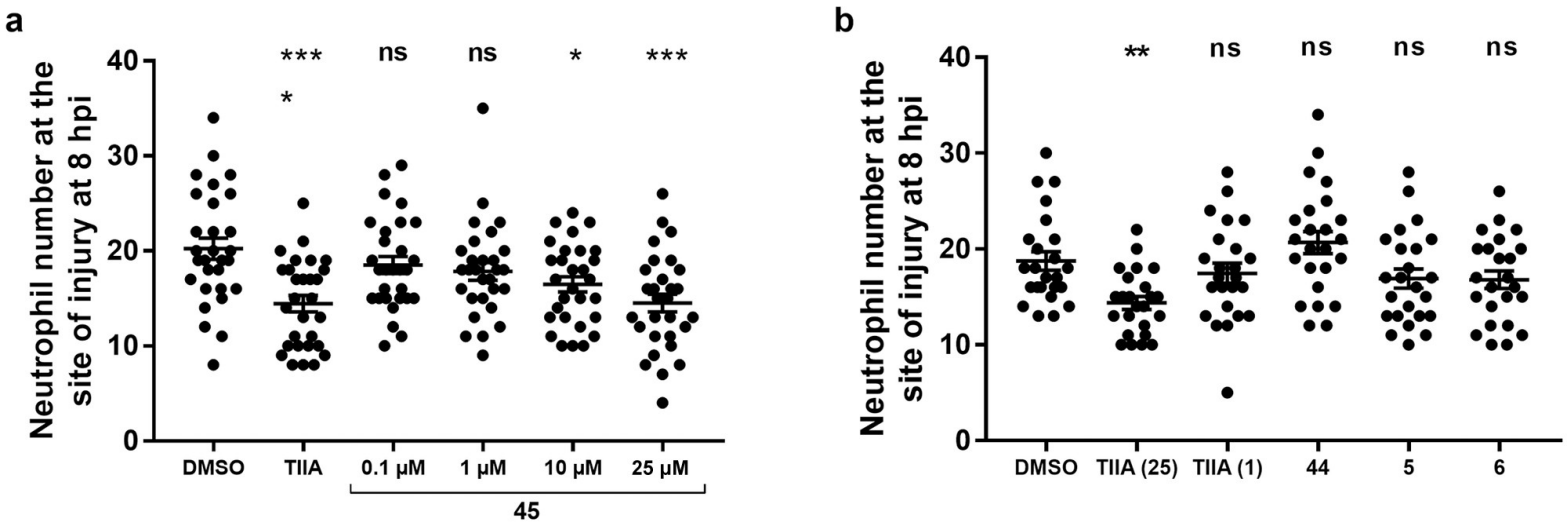
Effects of a) norlapachol 45 and b) lapachol 44, β-lapachone 5 and nor-β-lapachone 6 on resolution of neutrophilic inflammation. Tailfin transection was performed on transgenic zebrafish larvae, *TgBAC(mpx*:*GFP)i114* (3 dpf), and at 4 hpi, good responders were subjected to compound treatments: DMSO (0.5%), TIIA **2** (25 μM; also 1 μM for **b** only, as indicated in brackets), norlapachol **45** (**a**; 0.1, 1, 10, 25 μM as indicated) and lapachol **44**, β-lapachone **5** and nor-β-lapachone **6** (**b**; 1 μM). Neutrophil number at the site of injury was assessed at 8 hpi by counting GFP+ cells. Data shown as mean ± SEM; *n* = 24–29 larvae from 3–4 independent experiments per graph. * *P* < 0.05, ** *P* < 0.01, *** *P* < 0.001, **** *P* < 0.0001, ns not significant, in comparison to the DMSO control (one-way ANOVA with Dunnett’s multiple comparison post-test).

The same four compounds were evaluated for any effects on resolution of neutrophilic inflammation *in vivo* (Table 2, Fig 10). Norlapachol **45** was tested at a range of different concentrations from 0.1 to 25 μM, as the compound was not toxic to larvae at 25 μM in these experiments. The higher threshold for toxicity in resolution experiments may be attributed to the shorter treatment time with compound (4 hours instead of 6 hours in recruitment studies) and the fact that the tissue has been allowed to heal somewhat before application of compounds. Lapachol **44**, β-lapachone **5** and nor-β-lapachone **6** were each tested at 1 μM, as higher doses were toxic. TIIA **2** (25 μM) was used as a positive control, as in previous resolution experiments. Norlapachol **45** had no effect on the number of neutrophils at 0.1 and 1 μM (Fig 10a). However, a reduced neutrophil number at the site of injury was observed at 10 μM, and a highly significant reduction was seen at a concentration of 25 μM, although some of the larvae did show slow or absent circulation, possibly indicative of a toxic effect of norlapachol **45** at this concentration. Overall, the resolution data for norlapachol **45** show a good dose-response relationship. Finally, the remaining compounds lapachol **44**, β-lapachone **5**, and nor-β-lapachone **6** were analysed in similar inflammation resolution experiments, at 1 μM (Fig 10b). In these experiments, TIIA **2** was used at 25 μM (as the positive control) and 1 μM, again to compare the effects of this compound to the other compounds tested at the same concentration. However, none of these experimental compounds had any effect on resolution of neutrophilic inflammation.

Overall, norlapachol **45** affected both neutrophil recruitment to the site of injury and resolution at higher concentrations, yet at lower concentrations, only initial neutrophil recruitment was significantly affected (Table 2). Lapachol **44** exhibited no observable effect on either recruitment or resolution, and neither did nor-β-lapachone **6**. At a low concentration, β-lapachone **5**, affected only recruitment of neutrophils, and not resolution of neutrophilic inflammation. This may suggest a specific function of this compound (at this concentration) in earlier stages of the inflammatory response.

### Evaluation of the effects of active compounds on neutrophil apoptosis

**Fig 11:**
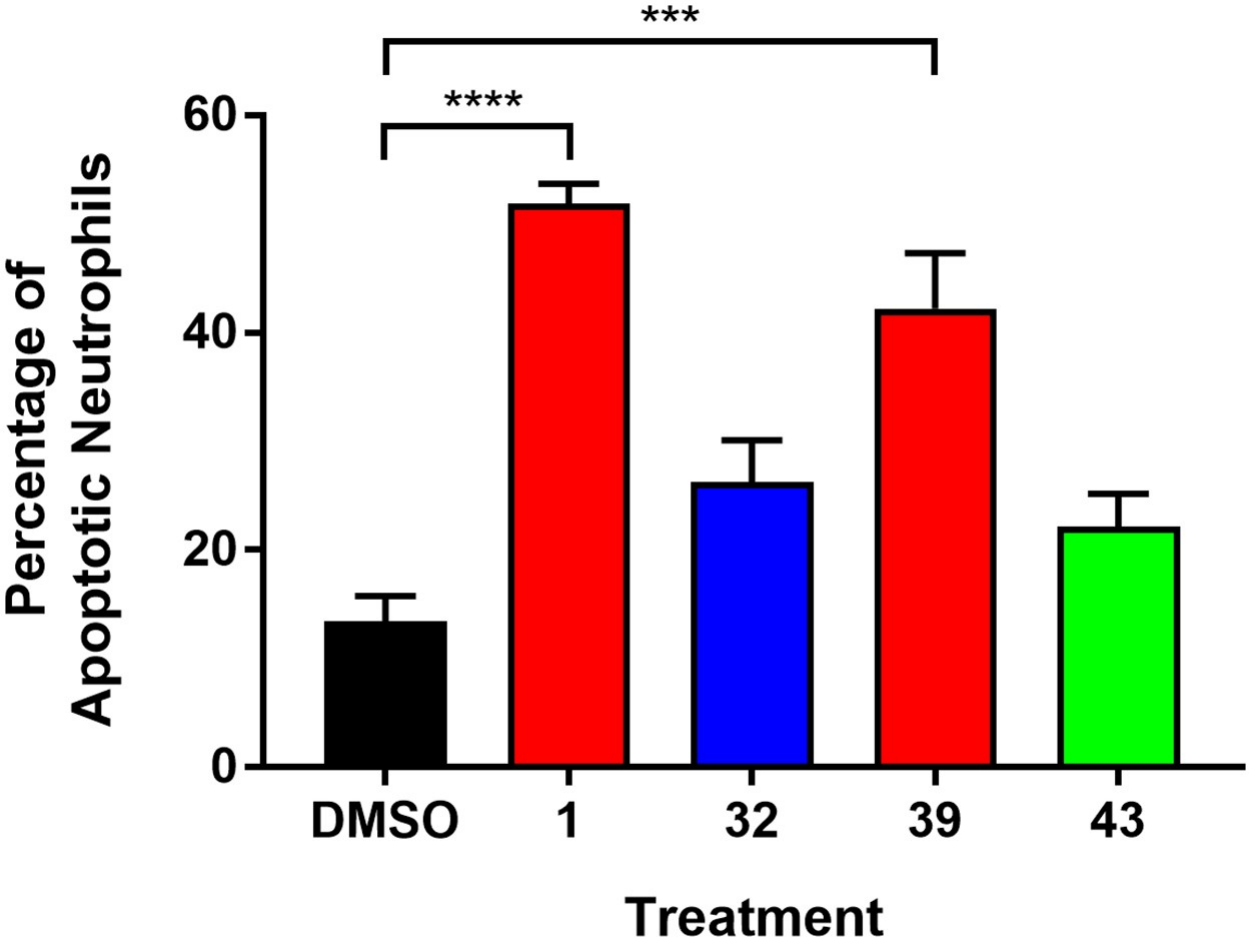
Effects of active compounds on neutrophil apoptosis. Freshly isolated human neutrophils were incubated for 6 hours at 37 ᵒC with the following compound treatments: DMSO, and compounds **1**, **32**, **39** and **43** (25 μM), with DMSO used as a vehicle control. Percentage neutrophil apoptosis for each condition was quantified from duplicate cytospins. Cells were classified as either apoptotic or non-apoptotic based on the morphology of their nuclei. Data shown as mean ± SEM; *n* = 4000 neutrophils from 4 independent experiments. *** *P* < 0.001, **** *P* < 0.0001, in comparison to the DMSO control (one-way ANOVA with Dunnett’s multiple comparison post-test).

Finally, to investigate effects on neutrophil structure and function directly, and begin to explore translational relevance, extending our results to a human system, the most active compounds *in vivo* were taken forward to evaluate their effects on neutrophil apoptosis. Freshly isolated human neutrophils were cultured for 6 hours with TI **1** and iso-TI **32**, both of which significantly affected resolution and recruitment of zebrafish neutrophils; and 6-methoxy-TI **39** and TI-acetal **43**, which accelerated inflammation resolution but had no effect on recruitment. The percentage of neutrophil apoptosis was assessed based on nuclear morphology. Quantification revealed that treatment with TI **1** and 6-methoxy-TI **39** resulted in a significant increase in apoptosis, whilst no such effect was observed for treatment with either iso-TI **32** or TI-acetal **43** (Fig 11). These data suggest that, at least for the structurally similar tanshinones **1** and **39**, neutrophil apoptosis substantially contributes to the anti-inflammatory activity of these compounds demonstrated in the zebrafish model. These data also correlate well with previous neutrophil apoptosis studies with the structurally similar TIIA **2**, which indicated that treatment with TIIA **2** resulted in an increase in neutrophil apoptosis, both *in vivo* in the zebrafish model, and *in vitro* with human neutrophils [63]. However, for the compounds lacking the *ortho*-quinone moiety, iso-TI **32** and TI-acetal **43**, neutrophil apoptosis appears to play a much smaller role in the anti-inflammatory effects of these compounds, suggesting either a different mechanism (such as reverse migration of neutrophils), or (perhaps more likely) differing relative contributions of multiple mechanisms, compared to the tanshinones.

## Conclusions

A small library of analogues and isomers of TI **1** was synthesised from commercially available starting materials using an optimised route, which allowed access to novel tanshinone derivatives as well as the much underexplored isotanshinones, about which little is known generally, including both chemically and biologically. Synthesis of an acetal variant of TI **1**, as well as structurally similar β-lapachones, was also undertaken. Evaluation of these compounds in a transgenic zebrafish model of inflammation revealed that various related molecules exhibited anti-inflammatory effects *in vivo*. Small changes in the molecular structures of these compounds resulted in varying effects on different stages of the inflammatory response, allowing for some broad SARs to be constructed. Three compounds– β-lapachone **5** and isotanshinones **36** and **38**—resulted in decreased neutrophil recruitment to the site of injury, but did not affect resolution of neutrophilic inflammation, whilst the methoxy-substituted tanshinone **39** accelerated inflammation resolution yet did not affect initial neutrophil recruitment. This compound is considered a particularly attractive candidate for potential pro-resolution therapeutics, as it exhibited an anti-inflammatory effect without affecting the recruitment of neutrophils to inflammatory sites. This compound also provides a basis for future studies, for example for future design and synthesis of molecular probes and other tool compounds. The acetal **43** also affected resolution but not neutrophil recruitment. Three compounds–tanshinones **1** and **35**, and isotanshinone **32**—reduced initial neutrophil recruitment and accelerated inflammation resolution. Furthermore, two of these compounds, tanshinones **1** and **39**, resulted in significant increases in apoptosis of human neutrophils. This study also revealed differences in the toxicity profiles between tanshinones and the closely related β-lapachones, exemplifying the utility of using zebrafish phenotypic screening in identifying safety issues with potential drug treatments early in the drug discovery and development process. Overall, this work has enabled an increased insight into the anti-inflammatory activities of various substituted tanshinones, isotanshinones, and structurally related molecules, working towards identification of candidates for clinical treatment of dysregulated inflammation which act specifically on inflammatory neutrophils.

## Experimental section

### Chemical synthesis

#### General reagents, materials and methods

All chemicals used were purchased from commercial suppliers and were used as received without further purification. Melting points were determined using a Gallenkamp melting point apparatus equipped with a thermometer. IR spectroscopy was performed on a PerkinElmer FT-IR Spectrum 65 or Spectrum 100 spectrometer, using either NaCl discs or a Universal diamond ATR. ^1^H, ^13^C and ^19^F NMR experiments were run on either a Bruker Avance 400 or Bruker Avance III HD 500 spectrometer at 298 K. Chemical shifts (δ) are reported in parts per million (ppm) relative to the deuterated lock solvent as an internal standard, where s = singlet, d = doublet, t = triplet, q = quartet, m = multiplet, br s = broad singlet, br d = broad doublet, br t = broad triplet, dd = doublet of doublets, ddd = double doublet of doublets, dt = doublet of triplets, td = triplet of doublets. All coupling constants are reported in hertz, Hz. Mass spectrometry was carried out on either an Agilent Technologies 6530 or 7200 spectrometer, using either electron impact (EI) or electrospray ionisation (ESI). TLC was performed on Merck silica gel 60 F_254_ aluminium-backed plates and visualised using ultraviolet light followed by staining with potassium permanganate dip. Column chromatography was carried out using silica gel obtained from VWR Chemicals, particle size 40–63 μm. Reactions using microwave conditions were carried out on a CEM Corporation Discover S-class microwave synthesiser, at pressure ≤ 17 bar and power ≤ 200 W. Synthesis of all intermediates for the tested compounds are provided in the S1 File.

#### General representative procedure A for reactions with chloroacetone to form tanshinones 1, 33, 35, 37, 39 and isotanshinones 32, 34, 36, 38, 40

A mixture of the alcohol **27–31** (100 mg, 0.42 mmol), ammonium acetate (32 mg, 0.42 mmol, 4 mmol g^-1^) and chloroacetone (10 mL g^-1^, 12 mmol) was heated in a sealed tube at 110 °C for 15 minutes under microwave conditions. The solution was cooled to room temperature, diluted with water (400 mL g^-1^) and extracted with DCM (3 x 400 mL g^-1^). The organic extracts were combined, dried (MgSO_4_), filtered and concentrated *in vacuo* to give the crude mixture which was purified by flash column chromatography (silica gel, DCM) to give the tanshinones **1, 33, 35, 37, 39** and isotanshinones **32, 34, 36, 38, 40**.

#### 1,6-Dimethylphenanthro[1,2-*b*]furan-10,11-dione, TI 1 and 4,8-dimethylphenanthro[3,2-*b*]furan-7,11-dione, iso-TI 32

General procedure **A** was followed, using the alcohol **27** (100 mg, 0.42 mmol) to give the dione **1** (21 mg, 12% over 3 steps) as a dark red solid; mp 232–234 °C (lit. [70] 229–230 °C); *δ*_H_(400 MHz; CDCl_3_) 9.23 (1 H, d, *J* 8.8, ArC*H*), 8.27 (1 H, d, *J* 8.8, ArC*H*), 7.77 (1 H, d, *J* 8.8, ArC*H*), 7.54 (1 H, dd, *J* 8.8, 7.1, ArC*H*), 7.34 (1 H, d, *J* 7.1, ArC*H*), 7.30 (1 H, s, OC*H*), 2.68 (3 H, s, C*H*_3_), 2.30 (3 H, d, *J* 0.8, C*H*_3_); *δ*_C_(100 MHz; CDCl_3_) 183.4 (*C* = O), 175.6 (*C* = O), 161.2 (Ar*C*), 142.0 (Ar*C*H), 135.2 (Ar*C*), 133.6 (Ar*C*), 132.9 (Ar*C*H), 132.7 (Ar*C*), 130.6 (Ar*C*H), 129.6 (Ar*C*), 128.3 (Ar*C*H), 124.8 (Ar*C*H), 123.1 (Ar*C*), 121.8 (Ar*C*), 120.5 (Ar*C*), 118.7 (Ar*C*H), 19.9 (*C*H_3_), 8.8 (*C*H_3_); *m/z* (ESI^+^) 299 (10%, M+Na^+^), 277 (100, M+H^+^). All data were in general agreement with the literature [66, 70, 71]. Also obtained was the dione **32** (22 mg, 12% over 3 steps) as an orange solid; mp 218–220 °C (lit. [69] 219–220 °C); ν_max_(ATR)/cm^-1^ 1655 (C = O), 1588 (C = O), 1533 (C = C); *δ*_H_(400 MHz; CDCl_3_) 9.66 (1 H, d, *J* 8.9, ArC*H*), 8.40 (1 H, dd, *J* 8.9, 0.8, ArC*H*), 8.32 (1 H, d, *J* 8.9, ArC*H*), 7.63 (1 H, dd, *J* 8.9, 7.0, ArC*H*), 7.54–7.52 (1 H, m, OC*H*), 7.48 (1 H, dt, *J* 7.0, 0.8, ArC*H*), 2.76 (3 H, s, ArC*H*_3_), 2.41 (3 H, d, *J* 1.2, C*H*_3_); *δ*_C_(101 MHz; CDCl_3_) 182.2 (*C* = O), 177.2 (*C* = O), 153.9 (Ar*C*), 145.2 (Ar*C*H), 136.0 (Ar*C*), 134.7 (Ar*C*), 133.5 (Ar*C*), 131.2 (Ar*C*), 130.9 (Ar*C*H), 129.8 (Ar*C*H), 129.3 (Ar*C*H), 127.2 (Ar*C*), 126.5 (Ar*C*), 126.2 (Ar*C*H), 122.1 (Ar*C*H), 120.8 (Ar*C*), 20.0 (*C*H_3_), 8.7 (*C*H_3_); *m/z* (ESI^+^) 299 (25%, M+Na^+^), 277 (100, M+H^+^). No ^13^C NMR spectroscopy data were reported in the literature; all other data were in general agreement with the literature [69, 70, 72].

#### 1-Methylphenanthro[1,2-*b*]furan-10,11-dione 33 and 8-methylphenanthro[3,2-*b*]furan-7,11-dione 34

General procedure **A** was followed, using the alcohol **28** (100 mg, 0.44 mmol) to give the dione **33** (24 mg, 15% over 3 steps) as a dark red solid; mp 229–233 °C (lit. [66] 226–229 °C); ν_max_(NaCl discs)/cm^-1^ 2922 (C-H), 2852 (C-H), 1669 (C = O), 1593 (C = C); *δ*_H_(400 MHz; CDCl_3_) 9.42 (1 H, d, *J* 8.7, ArC*H*), 8.12 (1 H, d, *J* 8.7, ArC*H*), 7.83 (2 H, br d, *J* 8.7, 2 x ArC*H*), 7.71 (1 H, br t, *J* 7.7, ArC*H*), 7.55 (1 H, t, *J* 7.7, ArC*H*), 7.33 (1 H, s, ArC*H*), 2.32 (3 H, s, C*H*_3_). No IR spectroscopy data were reported in the literature. ^1^H NMR data were in broad agreement with the literature [66], although precise chemical shift values were slightly shifted due to the different solvent used for analysis. Also obtained was the dione **34** (28 mg, 17% over 3 steps) as an orange solid; mp 199–202 °C; ν_max_(NaCl discs)/cm^-1^ 2921 (C-H), 1766 (C = O), 1664 (C = O), 1536 (C = C); *δ*_H_(400 MHz; CDCl_3_) 9.77 (1 H, d, *J* 8.5, ArC*H*), 8.28 (1 H, d, *J* 8.5, ArC*H*), 8.18 (1 H, d, *J* 8.5, ArC*H*), 7.90 (1 H, d, *J* 8.5, ArC*H*), 7.78–7.72 (1 H, m, ArC*H*), 7.68–7.61 (1 H, m, ArC*H*), 7.53 (1 H, s, ArC*H*), 2.40 (3 H, s, C*H*_3_); *δ*_C_(101 MHz; CDCl_3_) 182.2 (*C* = O), 177.2 (*C* = O), 153.7 (Ar*C*), 145.3 (Ar*C*H), 136.7 (Ar*C*), 135.1 (Ar*C*H), 134.0 (Ar*C*), 130.9 (Ar*C*), 130.1 (Ar*C*H), 128.7 (Ar*C*H), 128.5 (Ar*C*H), 128.0 (Ar*C*H), 127.0 (Ar*C*), 126.5 (Ar*C*), 122.3 (Ar*C*H), 120.9 (Ar*C*), 8.7 (*C*H_3_); *m/z* (EI^+^) 262.0622 (100%, M^+^ C_17_H_10_O_3_ requires 262.0624), 234 (42), 206 (40), 176 (36), 151 (15), 126 (8), 87 (7), 76 (8).

#### 1-Methyl-6-(trifluoromethyl)phenanthro[1,2-*b*]furan-10,11-dione 35 and 8-methyl-4-(trifluoromethyl)phenanthro[3,2-*b*]furan-7,11-dione 36

General procedure **A** was followed, using the unpurified alcohol **29** (435 mg) to give the dione **35** (6 mg, 1% over 3 steps) as a dark red solid; mp 204–208 °C; ν_max_(NaCl discs)/cm^-1^ 1674 (C = O), 1550 (C = C); *δ*_H_(400 MHz; CDCl_3_) 9.65 (1 H, d, *J* 8.9, ArC*H*), 8.50 (1 H, d, *J* 8.9, ArC*H*), 7.98 (1 H, d, *J* 8.9, ArC*H*), 7.94 (1 H, d, *J* 7.2 ArC*H*), 7.75 (1 H, br t, *J* 8.1, ArC*H*), 7.39 (1 H, br d, *J* 1.1, OC*H*), 2.33 (3 H, d, *J* 1.1, C*H*_3_); *δ*_C_(100 MHz; CDCl_3_) 183.3(*C* = O), 175.2 (*C* = O), 160.2 (Ar*C*), 142.8 (Ar*C*H), 133.0 (Ar*C*), 132.8 (q, *J*_C-F_ 3.0, Ar*C*H), 131.0 (Ar*C*H), 130.5 (Ar*C*), 129.8 (Ar*C*), 129.0 (Ar*C*H), 127.0 (q, *J*_C-F_ 30.3, Ar*C*), 126.0 (q, *J*_C-F_ 5.9, Ar*C*H), 124.2 (q, *J*_C-F_ 273.9, *C*F_3_), 123.1 (Ar*C*), 122.1 (Ar*C*), 121.2 (Ar*C*), 120.8 (Ar*C*H), 8.8 (*C*H_3_); *δ*_F_(377 MHz; CDCl_3_) -58.8; *m/z* (ESI^+^) 353 (18%, M+Na^+^), 331.0580 (100, M+H^+^ C_18_H_10_O_3_F_3_ requires 331.0577). Also obtained was the dione **36** (20 mg, 2% over 3 steps) as a dark brown solid; mp 183–186 °C; ν_max_(NaCl discs)/cm^-1^ 1661 (C = O), 1601 (C = O), 1536 (C = C); *δ*_H_(400 MHz; CDCl_3_) 10.07 (1 H, d, *J* 9.0, ArC*H*), 8.58 (1 H, d, *J* 9.0, ArC*H*), 8.45 (1 H, d, *J* 9.0, ArC*H*), 8.05 (1 H, d, *J* 7.1 ArC*H*), 7.79 (1 H, dd, *J* 9.0, 7.1, ArC*H*), 7.57 (1 H, q, *J* 1.1, OC*H*), 2.42 (3 H, d, *J* 1.1, C*H*_3_); *δ*_C_(100 MHz; CDCl_3_) 181.4 (*C* = O), 176.6 (*C* = O), 153.5 (Ar*C*), 145.7 (Ar*C*H), 134.0 (Ar*C*), 132.4 (Ar*C*H), 132.2 (Ar*C*), 131.6 (Ar*C*), 130.8 (q, *J*_C-F_ 2.7, Ar*C*H), 128.2 (Ar*C*H), 127.2 (Ar*C*), 127.0 (q, *J*_C-F_ 5.9, Ar*C*H), 126.7 (d, *J*_C-F_ 3.0, Ar*C*), 126.5 (Ar*C*), 124.3 (q, *J*_C-F_ 274.0, *C*F_3_), 124.0 (Ar*C*H), 121.0 (Ar*C*), 8.7 (*C*H_3_); *δ*_F_(377 MHz; CDCl_3_) -59.0; *m/z* (EI^+^) 330.0496 (90%, M^+^ C_18_H_9_O_3_F_3_ requires 330.0498), 261 [100, (M-CF_3_)^+^].

#### 6-Fluoro-1-methylphenanthro[1,2-*b*]furan-10,11-dione 37 and 4-fluoro-8-methylphenanthro[3,2-*b*]furan-7,11-dione 38

General procedure **A** was followed, using the unpurified alcohol **30** (140 mg) to give the dione **37** (18 mg, 2% over 3 steps) as a dark red solid; mp 187–191 °C; ν_max_(NaCl discs)/cm^-1^ 1675 (C = O), 1664 (C = O), 1594 (C = C); *δ*_H_(400 MHz; CDCl_3_) 9.20 (1 H, d, *J* 8.8, ArC*H*), 8.45 (1 H, d, *J* 8.8, ArC*H*), 7.89 (1 H, d, *J* 8.8, ArC*H*), 7.67–7.60 (1 H, m, ArC*H*), 7.36 (1 H, q, *J* 1.2, OC*H*), 7.22 (1 H, ddd, *J* 10.0, 7.8, 0.7, ArC*H*), 2.32 (3 H, d, *J* 1.2, C*H*_3_); *δ*_C_(100 MHz; CDCl_3_) 183.0 (*C* = O), 175.0 (*C* = O), 160.5 (Ar*C*), 158.8 (d, *J*_C-F_ 253.6, Ar*C*), 142.5 (Ar*C*H), 133.2 (d, *J*_C-F_ 2.5, Ar*C*), 131.0 (d, *J*_C-F_ 8.5, Ar*C*H), 130.9 (Ar*C*), 129.4 (d, *J*_C-F_ 7.3, Ar*C*H), 124.7 (d, *J*_C-F_ 15.4, Ar*C*), 122.5 (d, *J*_C-F_ 4.7, Ar*C*H), 122.3 (d, *J*_C-F_ 2.3, Ar*C*), 122.0 (Ar*C*), 121.0 (Ar*C*), 119.3 (d, *J*_C-F_ 1.6, Ar*C*H), 111.1 (d, *J*_C-F_ 19.3, Ar*C*H), 8.8 (*C*H_3_); *δ*_F_(377 MHz; CDCl_3_) -120.7; *m/z* (ESI^+^) 326 (34%, M+2Na^+^), 303 (20, M+Na^+^), 281.0612 (100, M+H^+^ C_17_H_10_O_3_F requires 281.0608). Also obtained was the dione **38** (12 mg, 1% over 3 steps) as a pale orange solid; mp 185–189 °C; ν_max_(NaCl discs)/cm^-1^ 1662 (C = O), 1591 (C = O), 1535 (C = C); *δ*_H_(400 MHz; CDCl_3_) 9.56 (1 H, d, *J* 8.9, ArC*H*), 8.50 (1 H, d, *J* 8.9, ArC*H*), 8.34 (1 H, d, *J* 8.9, ArC*H*), 7.71–7.64 (1 H, m, ArC*H*), 7.55 (1 H, d, *J* 1.0, OC*H*), 7.32 (1 H, dd, *J* 9.9, 7.8, ArC*H*), 2.41 (3 H, d, *J* 1.0, C*H*_3_); *δ*_C_(100 MHz; CDCl_3_) 181.8 (*C* = O), 176.7 (*C* = O), 158.4 (d, *J*_C-F_ 253.0, Ar*C*), 153.6 (Ar*C*), 145.5 (Ar*C*H), 134.5 (Ar*C*), 132.0 (d, *J*_C-F_ 2.8, Ar*C*), 130.0 (d, *J*_C-F_ 8.1, Ar*C*H), 127.5 (d, *J*_C-F_ 6.9, Ar*C*H), 127.1 (d, *J*_C-F_ 15.8, Ar*C*), 126.8 (d, *J*_C-F_ 2.6, Ar*C*), 126.7 (Ar*C*), 124.0 (d, *J*_C-F_ 4.6, Ar*C*H), 122.6 (d, *J*_C-F_ 1.6, Ar*C*H), 121.0 (Ar*C*), 112.1 (d, *J*_C-F_ 19.3, Ar*C*H), 8.7 (*C*H_3_); *δ*_F_(377 MHz; CDCl_3_) -120.9; *m/z* (EI^+^) 280.0532 (100%, M^+^ C_17_H_9_O_3_F requires 280.0530).

#### 6-Methoxy-1-methylphenanthro[1,2-*b*]furan-10,11-dione 39 and 4-methoxy-8-methylphenanthro[3,2-*b*]furan-7,11-dione 40

General procedure **A** was followed, using the alcohol **31** (100 mg, 0.39 mmol) to give the dione **39** (22 mg, 5% over 3 steps) as a dark brown solid; mp 249–253 °C; ν_max_(NaCl discs)/cm^-1^ 1670 (C = O), 1660 (C = O), 1587 (C = C), 1548 (C = C); *δ*_H_(400 MHz; CDCl_3_) 9.00 (1 H, d, *J* 8.8, ArC*H*), 8.66 (1 H, d, *J* 8.8, ArC*H*), 7.82 (1 H, d, *J* 8.8, ArC*H*), 7.62 (1 H, br t, *J* 8.4, ArC*H*), 7.34 (1 H, s, ArC*H*), 6.89 (1 H, d, *J* 7.8, ArC*H*), 4.04 (3 H, s, OC*H*_3_), 2.32 (3 H, s, C*H*_3_); *δ*_C_(126 MHz; CDCl_3_) 183.4 (*C* = O), 175.7 (*C* = O), 161.2 (Ar*C*), 155.7 (Ar*C*), 142.1 (Ar*C*H), 133.5 (Ar*C*), 131.5 (Ar*C*H), 131.2 (Ar*C*H), 130.5 (Ar*C*), 126.8 (Ar*C*), 122.4 (Ar*C*), 121.8 (Ar*C*), 120.7 (Ar*C*), 118.5 (Ar*C*H), 118.2 (Ar*C*H), 105.5 (Ar*C*H), 55.7 (O*C*H_3_), 8.8 (*C*H_3_); *m/z* (ESI^+^) 315 (11%, M+Na^+^), 293.0813 (100, M+H^+^ C_18_H_13_O_4_ requires 293.0808). Also obtained was the dione **40** (21 mg, 5% over 3 steps) as a red solid; mp 231–234 °C; ν_max_(ATR)/cm^-1^ 1662 (C = O), 1582 (C = O), 1535 (C = C); *δ*_H_(400 MHz; CDCl_3_) 9.32 (1 H, d, *J* 8.8, ArC*H*), 8.68 (1 H, d, *J* 8.8, ArC*H*), 8.24 (1 H, d, *J* 8.8, ArC*H*), 7.63 (1 H, dd, *J* 8.8, 7.9, ArC*H*), 7.51 (1 H, d, *J* 0.8, ArC*H*), 6.95 (1 H, d, *J* 7.9, ArC*H*), 4.04 (3 H, s, OC*H*_3_), 2.40 (3 H, d, *J* 0.8, C*H*_3_); *δ*_C_(101 MHz; CDCl_3_) 182.3 (*C* = O), 177.2 (*C* = O), 155.3 (Ar*C*), 153.9 (Ar*C*), 145.1 (Ar*C*H), 134.4 (Ar*C*), 132.0 (Ar*C*), 130.5 (Ar*C*H), 129.3 (Ar*C*), 129.2 (Ar*C*H), 126.51 (Ar*C*), 126.49 (Ar*C*), 121.6 (Ar*C*H), 120.8 (Ar*C*), 119.8 (Ar*C*H), 106.2 (Ar*C*H), 55.7 (O*C*H_3_), 8.8 (*C*H_3_); *m/z* (ESI^+^) 315 (43%, M+Na^+^), 293.0810 (100, M+H^+^ C_18_H_13_O_4_ requires 293.0808).

#### 1,6-Dimethylphenanthro[1,2-*b*]furan-10,11-dimethyldioxole, TI-acetal 43

TI **1** (27 mg, 0.10 mmol) was dissolved in anhydrous THF (7 mL) and stirred under a nitrogen atmosphere at room temperature. Sodium borohydride (4.0 mg, 0.10 mmol) was added in a single portion, and the solution stirred for 30 minutes. The solution was poured onto ice/water (20 mL), acidified with aqueous HCl solution (1 M, 3 mL), and extracted with DCM (3 x 20 mL). The organic extracts were combined, dried (MgSO_4_), filtered and concentrated *in vacuo* to give the crude diol **42** as an olive-green solid (29 mg), which was immediately dissolved in anhydrous toluene (10 mL). 2,2-Dimethoxypropane (0.025 mL, 0.20 mmol) and *para*-toluenesulfonic acid monohydrate (25 mg, 0.13 mmol) were added, and the reaction was stirred under a nitrogen atmosphere at reflux for 2 h. The mixture was cooled to room temperature, aqueous saturated NaHCO_3_ solution (20 mL) added, and extracted with diethyl ether (3 x 20 mL). The organic extracts were combined, dried (MgSO_4_), filtered and concentrated *in vacuo* to give an off-white solid (38 mg), which was purified twice by flash column chromatography (silica gel, DCM, and then silica gel, 19:1 40/60 petroleum ether/ethyl acetate) to give the acetal **43** as a beige solid (11 mg, 34%); mp 176–180 °C; ν_max_(ATR)/cm^-1^ 2917 (C-H); *δ*_H_(400 MHz; CDCl_3_) 9.13 (1 H, d, *J* 8.4, ArC*H*), 8.22 (1 H, d, *J* 9.2, ArC*H*), 7.89 (1 H, dd, *J* 9.2, 0.8, ArC*H*), 7.57–7.52 (2 H, m, 2 x ArC*H*), 7.45 (1 H, dt, *J* 7.1, 0.9, ArC*H*), 2.79 (3 H, s, ArC*H*_3_), 2.46 (3 H, d, *J* 1.3, ArC*H*_3_), 1.91 (6 H, s, 2 x C*H*_3_); *δ*_C_(126 MHz; CDCl_3_) 148.3 (Ar*C*), 141.4 (Ar*C*H), 138.2 (Ar*C*), 137.8 (Ar*C*), 134.1 (Ar*C*), 130.7 (Ar*C*), 128.9 (Ar*C*), 127.3 (Ar*C*H), 125.6 (Ar*C*H), 125.4 (Ar*C*H), 120.4 (Ar*C*H), 119.2 (Ar*C*H), 118.5 (Ar*C*), 114.5 (Ar*C*), 114.3 (Ar*C*), 113.0 (Ar*C*), 112.7 (Ar*C*), 26.1 (2 x *C*H_3_), 20.2 (Ar*C*H_3_), 9.2 (Ar*C*H_3_); *m/z* (ESI^+^) 319.1322 (100%, M+H^+^ C_21_H_19_O_3_ requires 319.1329), 383 (8), 359 (28), 261 (22).

#### β-Lapachone 5

Concentrated H_2_SO_4_ (1.0 mL) was added slowly to lapachol **44** (25 mg, 0.10 mmol), with stirring, until the solid completely dissolved. The solution was stirred for a further 5 minutes and poured onto ice (20 g). The precipitate was filtered off by vacuum filtration and washed with cold water (5 mL) to give an orange solid. The crude product was purified by flash column chromatography (silica gel, DCM– 19:1 DCM/MeOH) to give the dione **5** (21 mg, 85%) as an orange solid; mp 151–153 °C (lit. [82] 152–154 °C); *δ*_H_(400 MHz; CDCl_3_) 8.07 (1 H, dd, *J* 7.7, 1.0, ArC*H*), 7.83 (1 H, d, *J* 7.7, ArC*H*), 7.66 (1 H, td, *J* 7.7, 1.0, ArC*H*), 7.52 (1 H, td, *J* 7.7, 1.0, ArC*H*), 2.58 (2 H, t, *J* 6.7, C*H*_2_), 1.87 (2 H, t, *J* 6.7, C*H*_2_), 1.48 (6 H, s, 2 x C*H*_3_); *δ*_C_(101 MHz; CDCl_3_) 179.9 (*C* = O), 178.6 (*C* = O), 162.1 (Ar*C*), 134.8 (Ar*C*H), 132.6 (Ar*C*), 130.7 (Ar*C*H), 130.1 (Ar*C*), 128.6 (Ar*C*H), 124.1 (Ar*C*H), 112.7 (Ar*C*), 79.3 [*C*(CH_3_)_2_], 31.6 (*C*H_2_), 26.8 (2 x *C*H_3_), 16.2 (*C*H_2_). All data were in general agreement with the literature [82, 83].

#### Norlapachol 45

Lapachol **44** (150 mg, 0.62 mmol) was dissolved in THF (5 mL), sodium carbonate (72 mg, 0.68 mmol) in water (5 mL) was added, and the solution was heated to 60 °C. Aqueous hydrogen peroxide (30%, 1.0 mL, 13 mmol) was added and the solution was heated at 60 °C for 4 h. The reaction was cooled to rt, acidified with concentrated HCl (0.2 mL), and quenched with sodium sulfite (3.0 g). Aqueous NaOH (25%, 5 mL) and copper(II) sulfate (625 mg, 3.9 mmol) in water (5 mL) were added and the mixture was stirred at room temperature for 1.5 h. The solution was filtered through a pad of Celite, acidified with concentrated HCl (7 mL) and extracted with diethyl ether (3 x 75 mL). The organic extracts were combined, washed with brine (200 mL), dried (MgSO_4_), filtered and concentrated *in vacuo* to give an orange solid. The crude product was purified by recrystallisation from *n*-hexane to give the dione **45** (73 mg, 52%) as an orange solid; mp 123–125 °C (from *n*-hexane), (lit. [83] 121–122 °C); ν_max_(NaCl discs)/cm^-1^ 3364 (O-H), 2928 (C-H), 2910 (C-H), 1662 (C = O), 1645 (C = O), 1627 (C = C), 1593 (C = C); *δ*_H_(400 MHz; CDCl_3_) 8.15 (1 H, d, *J* 7.6, ArC*H*), 8.11 (1 H, d, *J* 7.6, ArC*H*), 7.78 (1 H, td, *J* 7.6, 1.1, ArC*H*), 7.71 (1 H, td, *J* 7.6, 1.1, ArC*H*), 7.55 (1 H, br s, O*H*), 6.02 (1 H, br s, C*H*), 2.01 (3 H, s, C*H*_3_), 1.70 (3 H, s, C*H*_3_); *δ*_C_(101 MHz; CDCl_3_) 184.8 (*C* = O), 181.6 (*C* = O), 151.2 (*C*), 143.6 (*C*), 134.9 (Ar*C*H), 133.0 (Ar*C*H), 132.9 (*C*), 129.5 (*C*), 126.9 (Ar*C*H), 126.1 (Ar*C*H), 120.9 (*C*), 113.7 (*C*H), 26.6 (*C*H_3_), 21.8 (*C*H_3_); *m/z* (ESI^+^) 251 (5%, M+Na^+^), 229.0862 (100, M+H^+^ C_14_H_13_O_3_ requires 229.0859). All data were in general agreement with the literature [83–85].

#### Nor-β-lapachone 6

Concentrated H_2_SO_4_ (1.5 mL) was added slowly to norlapachol **45** (30 mg, 0.13 mmol), with stirring, until the solid completely dissolved. The solution was stirred at room temperature for a further 5 minutes, poured onto ice (20 g) and rinsed with water (20 mL). The solution was extracted with DCM (3 x 40 mL), and the organic extracts were combined, washed with brine (100 mL), dried (MgSO_4_), filtered, and concentrated *in vacuo* to give an orange/pink solid. The crude product was purified by trituration with cold hexane (2 x 2 mL) and dried under high vacuum to give the dione **6** (29 mg, 95%) as an orange solid; mp 190–193 °C (lit. [86] 188–189 °C); *δ*_H_(400 MHz; CDCl_3_) 8.10 (1 H, d, *J* 7.6, ArC*H*), 7.68–7.64 (2 H, m, 2 x ArC*H*), 7.63–7.57 (1 H, m, ArC*H*), 2.97 (2 H, s, C*H*_2_), 1.63 (6 H, s, 2 x C*H*_3_); *δ*_C_(101 MHz; CDCl_3_) 181.4 (*C* = O), 175.7 (*C* = O), 168.8 (Ar*C*), 134.5 (Ar*C*H), 131.9 (Ar*C*H), 130.9 (Ar*C*), 129.3 (Ar*C*H), 127.9 (Ar*C*), 124.6 (Ar*C*H), 115.0 (Ar*C*), 93.8 [*C*(CH_3_)_2_], 39.3 (*C*H_2_), 28.4 (2 x *C*H_3_). All data were in general agreement with the literature [83, 86].

### Biological evaluation

#### Ethical permissions

Human neutrophil purification for this study was approved by the National Research Ethics Committee—Yorkshire & The Humber—Sheffield (05/Q2305/4). Fully informed written consent was obtained, and all clinical investigation was conducted according to the principles expressed in the Declaration of Helsinki. Zebrafish work in this study was approved by Sheffield University Animal Welfare and Ethical Review Body, Project Applications and Amendments Committee as part of the approval of the Home Office Project Licence PPL70/8178.

#### Reagents

All reagents were obtained from Sigma Aldrich/Merck (Darmstadt, Germany) unless stated otherwise. The c-Jun N-terminal kinase inhibitor SP600125 was obtained from StressMarq Biosciences (Victoria, Canada), and tanshinone IIA (TIIA) **2** was obtained from Generon (Slough, UK). All synthetic compounds were synthesised using chemistry as outlined in the previous section. All compounds were dissolved in dimethyl sulfoxide (DMSO).

#### Zebrafish husbandry

Zebrafish (*Danio rerio*) were raised and maintained in UK Home Office-approved aquaria at the Bateson Centre at The University Of Sheffield, in accordance with standard protocols and procedures [87]. Transgenic zebrafish larvae which express GFP specifically in neutrophils, *TgBAC(mpx*:*GFP)i114* [88], 3 days post-fertilisation (dpf), were used for all experiments.

#### Zebrafish inflammation assays

All compounds were diluted from the stock solutions and administered by immersion in aqueous solutions. DMSO (0.5% concentration) was used as the negative control. In neutrophil recruitment experiments, SP600125 (30 μM) or tanshinone I (TI) **1** (25 μM) was the positive control, whilst in inflammation resolution experiments, TIIA **2** (25 μM) was used.

Zebrafish larvae were injured by transection of the tailfin using a micro-scalpel blade, as posterior as possible in the region where the pigment pattern was disrupted, inducing an inflammatory response. Neutrophil counting was performed according to standard procedures used previously [63, 64, 88]. To assess neutrophil recruitment, zebrafish larvae were injured and treated immediately with test compounds at the stated concentrations. At 4 or 6 hours post-injury (hpi), larvae were anaesthetised and the number of neutrophils at the site of injury was counted. To study the resolution of neutrophilic inflammation, zebrafish larvae were injured and allowed to recover. Larvae that mounted a good inflammatory response (around 20–25 neutrophils at the site of injury; termed ‘good responders’) were selected for compound treatment at the stated concentrations at 4 hpi. At 8 hpi, larvae were anaesthetised and the number of neutrophils at the site of injury was counted. All experiments were performed blind to treatment groups.

#### Human neutrophil apoptosis experiments

Peripheral blood neutrophils were purified from healthy volunteers by dextran sedimentation and Percoll gradients as previously described [89]. Neutrophils were cultured at 37 ᵒC / 5% CO_2_ for 6 hours with either DMSO (negative control) or the compounds stated, in duplicate. Cytospins were produced with a cytocentrifuge at 300 rpm for 3 minutes for each condition. Cytospin slides were fixed with methanol then stained with Kwik-Diff (Thermo Scientific). Using an upright light microscope (Nikon) at 100x magnification, neutrophils were classified as either apoptotic or non-apoptotic based on the morphology of the nuclei. 1000 Neutrophils were counted per condition (500 per cytospin) to calculate percentage neutrophil apoptosis.

#### Statistical analysis

All statistical analysis was carried out using Prism 7.0 (GraphPad, San Diego, USA), version 7.00. For all experiments, at least three biological repeats were performed, from which data were pooled and then analysed. In all figures, data are shown as mean ± SEM of all data points from individual larvae or human neutrophils. Statistical analysis was performed using a one-way analysis of variance (ANOVA) test with Dunnett’s multiple comparison post-test for comparing treatment neutrophil numbers to those of the DMSO negative control.

## Supporting information

S1 File Supporting information. Chemical synthesis of compounds **8–9**, **11**, **13–31**; details of optimisation studies, including information on Design of Experiments (DoE) studies, and reactions involving methoxy-substituted compounds; and copies of ^1^H, ^13^C and ^19^F NMR spectra.
(DOCX)
