# Supplementary material for: Evaluation of the anti-inflammatory effects of synthesised tanshinone I and isotanshinone I analogues in zebrafish
Source: PLoS One. 2020 Oct 6;15(10):e0240231. doi: 10.1371/journal.pone.0240231 (PMC7537861; doi:10.1371/journal.pone.0240231)
Supplement: S1 File — Chemical synthesis of compounds 8–9, 11, 13–31; details of optimisation studies, including information on Design of Experiments (DoE) studies, and reactions involving methoxy-substituted compounds; and copies of 1H, 13C and 19F NMR spectra. (DOCX) [file pone.0240231.s001.docx]

**Evaluation of the anti-inflammatory effects of synthesised tanshinone I and isotanshinone I analogues in zebrafish**

Matthew J. Foulkes,^1,2,3^ Faith H. Tolliday,^2,3^ Katherine M. Henry,^2,3^ Stephen A. Renshaw,^2,3^ and Simon Jones^1*^

^1^ Department of Chemistry, The University of Sheffield, Sheffield, UK.

^2^ The Bateson Centre, The University of Sheffield, Sheffield, UK.

^3^ Department of Infection, Immunity & Cardiovascular Disease, The University of Sheffield, Sheffield, UK.

* Corresponding author

E-mail: simon.jones@sheffield.ac.uk (SJ)

**Supplementary Information (SI)**

Experimental - chemical synthesis of compounds **8-9**, **11**, **13-31**

Details of optimisation studies, including information on Design of Experiments (DoE) studies, and reactions involving methoxy-substituted compounds

NMR spectra of synthesised compounds

References

**Experimental**

**General reagents, materials and methods**

All chemicals used were purchased from commercial suppliers and were used as received without further purification. Melting points were determined using a Gallenkamp melting point apparatus equipped with a thermometer. IR spectroscopy was performed on a PerkinElmer FT-IR Spectrum 65 or Spectrum 100 spectrometer, using NaCl discs. ^1^H and ^13^C and NMR experiments were run on either a Bruker Avance 400 or Bruker Avance III HD 500 spectrometer at 298 K. Chemical shifts (δ) are reported in parts per million (ppm) relative to the deuterated lock solvent as an internal standard, where s = singlet, d = doublet, t = triplet, q = quartet, m = multiplet, br s = broad singlet, br d = broad doublet, br t = broad triplet, app d = apparent doublet, app t = apparent triplet, dd = doublet of doublets, ddd = double doublet of doublets, td = triplet of doublets, dq = doublet of quartets. All coupling constants are reported in hertz, Hz. TLC was performed on Merck silica gel 60 F_254_ aluminium-backed plates and visualised using ultraviolet light followed by staining with potassium permanganate dip. Column chromatography was carried out using silica gel obtained from VWR Chemicals, particle size 40-63 μm.

**2-Bromo-6-methoxy-1,4-hydroquinone 8**

Sodium percarbonate (18.71 g, 119.2 mmol) was added to a solution of 5-bromovanillin **7** (25.03 g, 108.4 mmol) in THF (300 mL) and water (120 mL) and stirred at room temperature for 5 h. The reaction was quenched with sodium sulfite (15.00 g), filtered, and concentrated *in vacuo*. The organic product was extracted with ethyl acetate (3 x 300 mL), dried (MgSO_4_), filtered and concentrated *in vacuo* to give the hydroquinone **8** (20.34 g, 86%) as a grey solid which was used without further purification; mp 142-146 °C (lit.[1] 139-141 °C); *δ*_H_[400 MHz; (CD_3_)_2_CO] 8.13 (1 H, s, O*H*), 7.57 (1H, s, O*H*), 6.59 (1 H, d, *J* 2.6, ArC*H*), 6.50 (1 H, d, *J* 2.6, ArC*H*), 3.82 (3 H, s, OC*H*_3_); *δ*_C_[100 MHz; (CD_3_)_2_CO] 150.7 (Ar*C*), 148.6 (Ar*C*), 137.3 (Ar*C*), 110.0 (Ar*C*H), 108.1 (Ar*C*), 99.6 (Ar*C*H), 55.6 (O*C*H_3_). All data were in agreement with the literature.[1]

**2-Bromo-6-methoxy-[1,4]-benzoquinone 9**

A solution of iron(III) chloride hexahydrate (125.4 g, 463.9 mmol) in water (600 mL) was added to a solution of 2-bromo-6-methoxy-1,4-hydroquinone **8** (20.32 g, 92.77 mmol) in methanol (80 mL) with stirring. The resulting mixture was stirred at room temperature for 5 h. The organic layer was separated and extracted with DCM (3 x 250 mL), combined, washed with water (500 mL) and brine (500 mL), dried (MgSO_4_), filtered and concentrated *in vacuo* to give the benzoquinone **9** (19.84 g, 99%) as an orange solid which was used without further purification; mp 152-156 °C (lit.[1] 160-162 °C); *δ*_H_(400 MHz; CDCl_3_) 7.23 (1 H, d, *J* 2.2, C*H*CO), 5.98 (1 H, d, *J* 2.2, C*H*CO), 3.88 (3 H, s, OC*H*_3_); *δ*_C_(100 MHz; CDCl_3_) 184.6 (*C*), 174.5 (*C*), 158.3 (*C*), 138.5 (=*C*H), 134.3 (*C*), 107.7 (=*C*H), 56.9 (O*C*H_3_). All NMR data were in agreement with the literature.[1]

**General representative procedure A for radical decarboxylative alkylation reactions to form bromides 11, 13-16**

A mixture of the quinone **9** (2.00 g, 9.26 mmol), carboxylic acid (11.1 mmol) and silver(I) phosphate (1.94 g, 4.63 mmol) in acetonitrile (70 mL) was stirred under a nitrogen atmosphere in the dark and heated at reflux. A solution of ammonium persulfate (4.23 g, 18.5 mmol) in water (70 mL) was added slowly over 20 minutes *via* dropping funnel, and the resulting mixture was stirred at reflux for 2.5 h. The solution was cooled to room temperature and poured onto ice (50 g). Aqueous NaOH (1 M, 10 mL) and water (50 mL) were added and the organic layer was separated and extracted with DCM (3 × 150 mL). The organic extracts were combined, washed with brine (400 mL), dried (MgSO_4_), filtered and concentrated *in vacuo* to give material which was purified by flash column chromatography (silica gel, DCM) to give the bromides **11, 13-16**.

**3-Bromo-5-methoxy-2-[2-(2-methylphenyl)ethyl]-2,5-cyclohexadiene-1,4-dione 11**

General procedure **A** was followed, using 3-(2-methylphenyl)propionic acid **10** (1.82 g), to give the bromide **11** (1.67 g, 54 %) as a yellow solid; mp 179-184 °C (lit.[2] mp 149-152 °C); *δ*_H_(400 MHz; CDCl_3_) 7.24-7.14 (4 H, m, 4 × ArC*H*), 5.99 (1 H, s, C*H*CO), 3.88 (3 H, s, OC*H*_3_), 2.98-2.92 (2 H, m, C*H*_2_), 2.82-2.76 (2 H, m, C*H*_2_), 2.45 (3 H, s, ArC*H*_3_); *δ*_C_(100 MHz; CDCl_3_) 183.7 (*C*=O), 174.8 (*C*=O), 158.3 (*C*), 149.0 (*C*), 138.6 (*C*), 136.2 (*C*), 133.2 (*C*), 130.4 (Ar*C*H), 129.1 (Ar*C*H), 126.6 (Ar*C*H), 126.2 (Ar*C*H), 107.4 (*C*HCO), 56.7 (O*C*H_3_), 32.0 (*C*H_2_), 31.3 (*C*H_2_), 19.3 (*C*H_3_). All NMR data were in agreement with the literature.[2]

**3-Bromo-5-methoxy-2-(2-phenylethyl)-2,5-cyclohexadiene-1,4-dione 13**

General procedure **A** was followed, but on a larger scale, using the quinone **9** (3.00 g, 13.9 mmol), 3-phenylpropionic acid **12** (2.50 g, 16.7 mmol) and silver(I) phosphate (4.82 g, 11.5 mmol) in acetonitrile (150 mL), and ammonium persulfate (6.34 g, 27.8 mmol) in water (100 mL), to give the bromide **13** (2.04 g, 46%) as a yellow solid; mp 138-141 °C (lit.[2] 138-140 °C); *δ*_H_(400 MHz; CDCl_3_) 7.36-7.22 (5 H, m, 5 x ArC*H*), 5.98 (1 H, s, C*H*CO), 3.87 (3 H, s, OC*H*_3_), 3.03-2.97 (2 H, m, C*H*_2_), 2.83-2.77 (2 H, m, C*H*_2_); *δ*_C_(100 MHz; CDCl_3_) 183.6 (*C*=O), 174.8 (*C*=O), 158.3 (*C*), 149.0 (*C*), 140.4 (*C*), 133.3 (*C*), 128.55 (2 x Ar*C*H), 128.49 (2 x Ar*C*H), 126.5 (Ar*C*H), 107.4 (=*C*H), 56.7 (O*C*H_3_), 33.8 (*C*H_2_), 33.2 (*C*H_2_). All data were in agreement with the literature.[2]

**3-Bromo-5-methoxy-2-[2-(trifluoromethyl)phenethyl]-2,5-cyclohexadiene-1,4-dione 14**

General procedure **A** was followed, using 3-[2-(trifluoromethyl)phenyl]propionic acid (2.42 g), to give the bromide **14** (1.35 g, 38%) as a yellow solid; mp 144-147 °C (lit.[2] 143-145 °C); *δ*_H_(400 MHz; CDCl_3_) 7.66 (1 H, d, *J* 7.6, ArC*H*), 7.52 (1 H, t, *J* 7.6, ArC*H*), 7.46 (1 H, d, *J* 7.6, ArC*H*), 7.35 (1 H, t, *J* 7.6, ArC*H*), 6.00 (1 H, s, C*H*CO), 3.88 (3 H, s, OC*H*_3_), 3.08-2.95 (4 H, m, 2 x C*H*_2_); *δ*_C_(100 MHz; CDCl_3_) 183.5 (*C*=O), 174.7 (*C*=O), 158.3 (*C*), 148.4 (*C*), 139.0 (*C*), 133.6 (*C*), 131.9 (Ar*C*H), 131.4 (Ar*C*H), 128.6 (q, *J*_C-F_ 30.0, Ar*C*), 126.6 (Ar*C*H), 126.0 (q, *J*_C-F_ 5.7, Ar*C*H), 124.5 (q, *J*_C-F_ 273.6, *C*F_3_), 107.4 (=*C*H), 56.7 (O*C*H_3_), 32.8 (*C*H_2_), 30.3 (*C*H_2_). All data were in agreement with the literature.[2]

**3-Bromo-2-(2-fluorophenethyl)-5-methoxy-2,5-cyclohexadiene-1,4-dione 15**

General procedure **A** was followed, using 3-(2-fluorophenyl)propionic acid (1.87 g), to give the bromide **15** (1.44 g, 46%) as a yellow solid; mp 144-148 °C (lit.[2] 134-137 °C); *δ*_H_(400 MHz; CDCl_3_) 7.25-7.19 (2 H, m, 2 x ArC*H*), 7.11-6.99 (2 H, m, 2 x ArC*H*), 5.97 (1 H, s, C*H*CO), 3.87 (3 H, s, OC*H*_3_), 3.06-2.99 (2 H, m, C*H*_2_), 2.92-2.85 (2 H, m, C*H*_2_); *δ*_C_(100 MHz; CDCl_3_) 183.6 (*C*=O), 174.8 (*C*=O), 161.2 (d, *J*_C-F_ 245.4, Ar*C*F), 158.2 (*C*), 148.6 (*C*), 133.5 (*C*), 130.8 (d, *J*_C-F_ 4.6, Ar*C*H), 128.3 (d, *J*_C-F_ 8.1, Ar*C*H), 127.1 (d, *J*_C-F_ 16.2, Ar*C*), 124.1 (d, *J*_C-F_ 3.4, Ar*C*H), 115.3 (d, *J*_C-F_ 22.0, Ar*C*H), 107.3 (=*C*H), 56.7 (O*C*H_3_), 31.5 (*C*H_2_), 27.2 (*C*H_2_). All NMR data were in agreement with the literature.[2]

**3-Bromo-5-methoxy-2-(2-methoxyphenethyl)-2,5-cyclohexadiene-1,4-dione 16**

General procedure **A** was followed, using 3-(2-methoxyphenyl)propionic acid (2.00 g), to give an inseparable mixture of the bromide **16** and the benzoquinone **9** (1.07 g) as a yellow solid in a 60:40 ratio which was used without further purification. Analysis of the ^1^H NMR spectrum of this mixture indicated a 33% yield of the bromide **16**. A sample of this mixture was further purified for analytical purposes to give the bromide **16** as a yellow solid; mp 160-163 °C (lit.[2] 161-163 °C); *δ*_H_(400 MHz; CDCl_3_) 7.22 (1 H, td, *J* 7.7, 1.6, ArC*H*), 7.15 (1 H, dd, *J* 7.7, 1.6, ArC*H*), 6.89 (1 H, td, *J* 7.7, 0.7, ArC*H*), 6.84 (1 H, d, *J* 7.7, ArC*H*), 5.96 (1 H, s, C*H*CO), 3.86 (3 H, s, OC*H*_3_), 3.84 (3 H, s, OC*H*_3_), 3.04-2.98 (2 H, m, C*H*_2_), 2.88-2.82 (2 H, m, C*H*_2_); *δ*_C_(100 MHz; CDCl_3_) 183.7 (*C*=O), 174.9 (*C*=O), 158.1 (*C*), 157.6 (*C*), 149.6 (*C*), 132.9 (*C*), 130.1 (Ar*C*H), 128.6 (*C*), 127.8 (Ar*C*H), 120.5 (Ar*C*H), 110.2 (Ar*C*H), 107.3 (Ar*C*H), 56.7 (O*C*H_3_), 55.3 (O*C*H_3_), 31.3 (*C*H_2_), 28.7 (*C*H_2_). All data were in general agreement with the literature.[2]

**General representative procedure B for Heck reactions to form diones 17-26**

A solution of the bromide **11, 13-16** (2.24 mmol), palladium(II) acetate (25 mg, 0.111 mmol), triphenylphosphine (59 mg, 0.225 mmol) and potassium carbonate (930 mg, 6.73 mmol) in degassed toluene (80 mL mmol^-1^) was heated at reflux in the dark for 17 h under a nitrogen atmosphere, with stirring. The mixture was cooled to room temperature, concentrated *in vacuo*, diluted with water (40 mL mmol^-1^), and extracted with DCM (3 x 40 mL mmol^-1^). The organic extracts were combined, washed with brine (80 mL mmol^-1^), dried (MgSO_4_), filtered and concentrated *in vacuo* to give material which was separated from major impurities by flash column chromatography (silica gel, DCM) to give an inseparable mixture of the fully aromatised and non-aromatised diones **17-26**.

**3-Methoxy-8-methylphenanthrene-1,4-dione 17 and 9,10-dihydro-3-methoxy-8-methylphenanthrene-1,4-dione 18**

General procedure **B** was followed, using the bromide **11** (750 mg, 2.24 mmol), to give a bright orange solid (378 mg, 67%) as an inseparable mixture of diones **17** and **18** in a 75:25 ratio which was used without further purification. Selected ^1^H NMR data from product mixture corresponding to the dione **17**: *δ*_H_(400 MHz; CDCl_3_) 9.42 (1 H, d, *J* 8.8, ArC*H*), 8.43 (1 H, br d, *J* 8.8, ArC*H*), 8.25 (1 H, br d, *J* 8.8, ArC*H*), 7.65 (1 H, dd, *J* 8.8, 6.9, ArC*H*), 7.50 (1 H, br d, *J* 6.9, ArC*H*), 6.17 (1 H, br s, C*H*CO), 3.97 (3 H, br s, OC*H*_3_), 2.77 (3 H, s, C*H*_3_). All data were in general agreement with the literature.[3] Selected ^1^H NMR data from product mixture corresponding to the dione **18**: *δ*_H_(400 MHz; CDCl_3_) 7.92-7.86 (1 H, app t, *J* 4.5, ArC*H*), 7.23 (2 H, d, *J* 4.5, ArC*H*), 5.99 (1 H, br s, C*H*CO), 3.88 (3 H, br s, OC*H*_3_), 2.79-2.74 (2 H, m, C*H*_2_), 2.74-2.70 (2 H, m, C*H*_2_), 2.36 (3 H, s, C*H*_3_). No data were reported in the literature.

**3-Methoxyphenanthrene-1,4-dione 19 and 9,10-dihydro-3-methoxyphenanthrene-1,4-dione 20**

General procedure **B** was followed, using the bromide **13** (200 mg, 0.622 mmol), to give a pale red solid (109 mg, 73%) as an inseparable mixture of diones **19** and **20** in a 96:4 ratio which was used without further purification. Selected ^1^H NMR data from product mixture corresponding to the dione **19**: *δ*_H_(400 MHz; CDCl_3_) 9.55 (1 H, d, *J* 8.8, ArC*H*), 8.20 (2 H, app d, *J* 1.2, 2 x ArC*H*), 7.92 (1 H, d, *J* 8.1, ArC*H*), 7.77 (1 H, ddd, *J* 8.8, 6.8, 1.2, ArC*H*), 7.66 (1 H, ddd, *J* 8.1, 6.8, 1.2, ArC*H*), 6.17 (1 H, s, C*H*CO), 3.96 (3 H, s, OC*H*_3_). All data were in general agreement with the literature.[4,5] Selected ^1^H NMR data from product mixture corresponding to the dione **20**: *δ*_H_(400 MHz; CDCl_3_) 5.99 (1 H, s, C*H*CO), 3.88 (3 H, s, OC*H*_3_), 2.83-2.78 (2 H, m, C*H*_2_), 2.76-2.72 (2 H, m, C*H*_2_). No data were reported in the literature.

**3-Methoxy-8-(trifluoromethyl)phenanthrene-1,4-dione 21 and 9,10-dihydro-3-methoxy-8-trifluoromethyl)phenanthrene-1,4-dione 22**

General procedure **B** was followed, using the bromide **14** (1.35 g, 3.47 mmol), to give a dark orange solid (500 mg) as an inseparable mixture including diones **21** and **22** in a 65:35 ratio alongside an additional unidentified compound, which was used without further purification. Selected ^1^H NMR peaks from product mixture corresponding to the dione **21**: *δ*_H_(400 MHz; CDCl_3_) 9.81 (1 H, d, *J* 8.9, ArC*H*), 8.59 (1 H, dq, *J* 8.9, 0.9, ArC*H*), 8.37 (1 H, d, *J* 8.9, ArC*H*), 8.06 (1 H, d, *J* 7.2, ArC*H*), 7.86-7.81 (1 H, m, ArC*H*), 6.23 (1 H, s, C*H*CO), 3.99 (3 H, s, OC*H*_3_). Selected ^1^H NMR peaks from product mixture corresponding to the dione **22**: *δ*_H_(400 MHz; CDCl_3_) 5.97 (1 H, s, C*H*CO), 3.85 (3 H, s, OC*H*_3_), 3.04-2.97 (2 H, m, C*H*_2_), 2.80-2.74 (2 H, m, C*H*_2_). Selected ^1^H NMR peaks from product mixture corresponding to the unidentified compound: *δ*_H_(400 MHz; CDCl_3_) 6.01(1 H, s, =C*H*), 3.89 (3 H, s, OC*H*_3_). No data were reported in the literature.

**8-Fluoro-3-methoxyphenanthrene-1,4-dione 23 and 9,10-dihydro-8-fluoro-3-methoxyphenanthrene-1,4-dione 24**

General procedure **B** was followed, using the bromide **15** (1.34 g, 3.95 mmol), to give a dark orange solid (197 mg) as an inseparable mixture including diones **23** and **24** in an 80:20 ratio alongside an additional unidentified compound, which was used without further purification. Selected ^1^H NMR peaks from product mixture corresponding to the dione **23**: *δ*_H_(400 MHz; CDCl_3_) 9.35 (1 H, d, *J* 8.8, ArC*H*), 8.54 (1 H, d, *J* 8.8, ArC*H*), 8.29 (1 H, d, *J* 8.8, ArC*H*), 7.74-7.67 (1 H, m, ArC*H*), 7.37-7.32 (1 H, m, ArC*H*), 6.21 (1 H, s, C*H*CO), 3.98 (3 H, s, OC*H*_3_). Selected ^1^H NMR peaks from product mixture corresponding to the dione **24**: *δ*_H_(400 MHz; CDCl_3_) 5.95 (1 H, s, =C*H*), 3.84 (3 H, s, OC*H*_3_), 2.93-2.87 (2 H, m, C*H*_2_), 2.80-2.73 (2 H, m, C*H*_2_). Selected ^1^H NMR peaks from product mixture corresponding to the unidentified compound: *δ*_H_(400 MHz; CDCl_3_) 6.90 (1 H, s, ArC*H*), 5.99 (1 H, s, C*H*CO), 3.88 (3 H, s, OC*H*_3_). No data were reported in the literature.

**3,8-Dimethoxyphenanthrene-1,4-dione 25 and 9,10-dihydro-3,8-dimethoxyphenanthrene-1,4-dione 26**

General procedure **B** was followed, using the bromide **16** (1.02 g, 2.91 mmol), to give a red solid (218 mg, 28%) as an inseparable mixture of diones **25** and **26** in an 82:18 ratio which was used without further purification. Selected ^1^H NMR data from product mixture corresponding to the dione **25**: *δ*_H_(400 MHz; CDCl_3_) 9.10 (1 H, d, *J* 8.8, ArC*H*), 8.71 (1 H, d, *J* 8.8, ArC*H*), 8.18 (1 H, d, *J* 8.8, ArC*H*), 7.66 (1 H, br t, *J* 8.4, ArC*H*), 6.98 (1 H, d, *J* 7.7, ArC*H*), 6.15 (1 H, s, C*H*CO), 4.04 (3 H, s, OC*H*_3_), 3.95 (3 H, s, OC*H*_3_). All data were in agreement with the literature.[5] Selected ^1^H NMR data from product mixture corresponding to the dione **26**: *δ*_H_(400 MHz; CDCl_3_) 5.94 (1 H, s, C*H*CO), 3.83 (3 H, s, OC*H*_3_), 3.80 (3 H, s, OC*H*_3_), 2.88-2.82 (2 H, m, C*H*_2_), 2.77-2.72 (2 H, m, C*H*_2_). No data were reported in the literature.

**General representative procedure C for demethylation to form alcohols 27-31**

Aqueous NaOH (2 M, 50 mL g^-1^, 40.0 mmol) was added to a mixture of the ethers **17-26** (378 mg, 1.50 mmol) in ethanol (50 mL g^-1^), and the resulting mixture was stirred at reflux for 1 h. The solution was cooled to room temperature, acidified with aqueous HCl (1 M, 100 mL g^-1^), water was added (50 mL g^-1^), and the solution was extracted with ethyl acetate (3 × 63 mL g^-1^). The organic extracts were combined, washed with brine (250 mL g^-1^), dried (MgSO_4_), filtered and concentrated *in vacuo* to give the alcohols **27-31**.

**3-Hydroxy-8-methylphenanthrene-1,4-dione 27**

General procedure **C** was followed, using the mixture of diones **17** and **18** (378 mg, 1.50 mmol) to give the alcohol **27** (347 mg, 97%) as a pale red solid that did not require further purification; mp 208-214 °C (lit.[2] mp 209-212 °C); *δ*_H_[400 MHz; (CD_3_)_2_SO] 11.67 (1 H, br s, ArO*H*), 9.32 (1 H, d, *J* 8.8, ArC*H*), 8.51 (1 H, d, *J* 8.8, ArC*H*), 8.12 (1 H, d, *J* 8.8, ArC*H*), 7.69 (1 H, dd, *J* 8.8, 7.0, ArC*H*), 7.57 (1 H, d, *J* 7.0, ArC*H*), 6.15 (1 H, s, C*H*CO), 2.72 (3 H, s, ArC*H*_3_); *δ*_C_[100 MHz; (CD_3_)_2_SO] 185.5 (*C*=O), 184.3 (*C*=O), 160.6 (*C*), 135.6 (*C*), 135.1 (*C*), 132.6 (*C*), 132.1 (Ar*C*H), 130.4 (Ar*C*H), 130.1 (*C*), 129.4 (Ar*C*H), 125.8 (*C*), 125.2 (Ar*C*H), 121.7 (Ar*C*H), 108.4 (Ar*C*H), 19.9 (*C*H_3_). All data were in general agreement with the literature.[2]

**3-Hydroxyphenanthrene-1,4-dione 28**

General procedure **C** was followed, using the mixture of diones **19** and **20** (222 mg, 0.931 mmol) to give the alcohol **28** (200 mg, 97%) as a dark orange solid which was used without further purification; mp 199-202 °C (lit.[6] 200 °C); *δ*_H_[400 MHz; (CD_3_)_2_CO] 9.83 (1 H, br s, O*H*), 9.58 (1 H, d, *J* 8.7, ArC*H*), 8.41 (1 H, d, *J* 8.7, ArC*H*), 8.19 (1 H, dd, *J* 8.7, 1.7, ArC*H*), 8.10 (1 H, d, *J* 8.2, ArC*H*), 7.86-7.80 (1 H, m, ArC*H*), 7.77-7.71 (1 H, m, ArC*H*), 6.25 (1 H, d, *J* 1.7, C*H*CO). All ^1^H NMR data were in broad agreement with the literature,[2] although precise values were slightly shifted due to the different solvent used for analysis.

**3-Hydroxy-8-(trifluoromethyl)phenanthrene-1,4-dione 29**

General procedure **C** was followed, using the mixture of diones **21** and **22** (500 mg) to give the crude alcohol **29** (447 mg) as an orange solid which could not be purified further by either flash column chromatography or recrystallisation. Selected peaks from ^1^H NMR spectrum of crude material corresponding to the alcohol **29**: *δ*_H_[400 MHz; (CD_3_)_2_CO] 9.89 (1 H, d, *J* 9.0, ArC*H*), 8.64 (1 H, br d, *J* 9.0, ArC*H*), 8.40 (1 H, d, *J* 9.0, ArC*H*), 8.21 (1 H, d, *J* 7.3, ArC*H*), 8.00-7.94 (1 H, m, ArC*H*), 6.31 (1 H, s, C*H*CO). All ^1^H NMR data were in broad agreement with the literature,[2] although precise values were slightly shifted due to the different solvent used for analysis.

**8-Fluoro-3-hydroxyphenanthrene-1,4-dione 30**

General procedure **C** was followed, using the mixture of diones **23** and **24** (194 mg) to give the crude alcohol **30** (170 mg) as a dark red solid which could not be purified further by either flash column chromatography or recrystallisation. Selected peaks from ^1^H NMR spectrum of crude material corresponding to the alcohol **30**: *δ*_H_[400 MHz; (CD_3_)_2_CO] 9.95 (1 H, br s, O*H*), 9.40 (1 H, d, *J* 8.8, ArC*H*), 8.60 (1 H, d, *J* 8.8, ArC*H*), 8.29 (1 H, d, *J* 8.8, ArC*H*), 7.86-7.79 (1 H, m, ArC*H*), 7.51 (1 H, ddd, *J* 10.4, 7.8, 0.7, ArC*H*), 6.28 (1 H, s, C*H*CO). All ^1^H NMR data were in broad agreement with the literature,[2] although precise values were slightly shifted due to the different solvent used for analysis.

**3-Hydroxy-8-methoxyphenanthrene-1,4-dione 31**

General procedure **C** was followed, using the mixture of diones **25** and **26** (218 mg, 0.813 mmol) to give the alcohol **31** (192 mg, 93%) as a dark red solid which was used without further purification; mp 204-207 °C (lit.[2] 185-187 °C); ν_max_(NaCl discs)/cm^-1^ 3281 (O-H), 1657 (C=O), 1634 (C=O), 1582 (C=C); *δ*_H_[400 MHz; (CD_3_)_2_SO] 11.65 (1 H, br s, O*H*), 8.97 (1 H, d, *J* 8.8, ArC*H*), 8.59 (1 H, d, *J* 8.8, ArC*H*), 8.02 (1 H, d, *J* 8.8, ArC*H*), 7.70 (1 H, t, *J* 7.8, ArC*H*), 7.15 (1 H, d, *J* 7.8, ArC*H*), 6.13 (1 H, s, C*H*CO), 4.00 (3 H, s, OC*H*_3_). IR spectroscopy data were not reported in the literature. All other spectroscopic data were in agreement with the literature.[2]

**Details of optimisation studies**

**Optimisation of the radical decarboxylative coupling**

Initially employing conditions identified by the literature as being optimal provided the desired alkylation product **11**, but in a significantly lower yield of around 20%, in comparison to yields of 65-79% previously reported.[2] Variation of several reaction parameters was then explored, including: carrying the reaction out in the dark; slightly modifying the work-up procedure by adding ice and reducing the volume of aqueous sodium hydroxide used; and adding the oxidant as a more dilute solution. Each modification separately led to a slightly increased yield (25-42% yield). Interestingly, reducing the number of equivalents of carboxylic acid **10** from 2 to 1.2 had no detrimental effect on the yield (35%).

**Design of Experiments (DoE) studies for the radical decarboxylative coupling**

Additional optimisation studies were also carried out on the radical decarboxylative coupling step. Using the unsubstituted 3-phenylpropionic acid **12** as a model substrate (Fig 1), to eliminate the possibility of an additional substituent having any electronic or steric impact on the reaction, a number of reaction parameters were investigated using a Design of Experiments (DoE) approach (summarised in Table 1). Using the Custom Design function in JMP software (JMP®, Version 12. SAS Institute Inc., Cary, NC, 1989-2007), the single most important factor was determined to be the method of aqueous persulfate addition. Adding the persulfate solution *via* glass dropping funnel gave a consistently elevated yield compared to addition *via* metal syringe, an observation noted elsewhere with aqueous persulfate solutions,[7] thought to be a result of the metal syringe accelerating the rate of decomposition of the oxidant solution.[8] Using silver(I) phosphate gave higher yields than other silver(I) salts investigated, whilst heating at 85 °C and increasing the number of equivalents of silver(I) salt both gave mild increases in product yield, with all other changes identified as having minimal or no effect.

**Fig 1. General radical alkylation reaction for optimisation studies using DoE.**

**Table 1**. **The ten radical alkylation experiments carried out, as determined by the JMP DoE software.**

| **Eq. acid 12** | **Eq. Ag(I) salt** | **Ag(I) salt** | **Vol. MeCN / mL** | **Vol. H_2_O / mL** | **Time / h** | **Temp. / °C** | **Metal needle used?** | **% yield product 13*** |
| --- | --- | --- | --- | --- | --- | --- | --- | --- |
| 0.5 | 1 | Ag_3_PO_4_ | 3 | 5 | 0.5 | 40 | No | 25% |
| 0.5 | 0.1 | AgNO_3_ | 3 | 5 | 0.5 | 85 | Yes | 10% |
| 0.5 | 0.1 | Ag_3_PO_4_ | 15 | 10 | 0.5 | 85 | Yes | 11% |
| 0.5 | 1 | AgNO_3_ | 3 | 10 | 3 | 40 | Yes | 18% |
| 0.5 | 1 | Ag_2_CO_3_ | 15 | 5 | 3 | 85 | No | 26% |
| 2 | 0.1 | Ag_2_CO_3_ | 3 | 10 | 0.5 | 40 | No | 6% |
| 2 | 1 | Ag_2_CO_3_ | 15 | 5 | 0.5 | 40 | Yes | 0% |
| 2 | 1 | AgNO_3_ | 15 | 10 | 0.5 | 85 | No | 51% |
| 2 | 0.1 | AgNO_3_ | 15 | 5 | 3 | 40 | No | 18% |
| 2 | 1 | Ag_3_PO_4_ | 3 | 5 | 3 | 85 | Yes | 24% |

* Represents isolated product after chromatographic purification.

**Formation of the radical decarboxylative coupling product 16**

For the radical decarboxylative coupling product with the methoxy substituent **16**, some benzoquinone starting material **9** remained, which could not be separated from the desired alkylation product **16** by either chromatographic purification on silica, or recrystallisation. Analysis of the ^1^H NMR spectrum of the mixture indicated a 60:40 ratio of the alkylation product **16** and the starting material **9**, corresponding to an approximate 33% overall yield of desired product **16**.

**Optimisation of the intramolecular Heck reaction**

Attempted optimisation of the intramolecular Heck reaction using the unsubstituted variant **13** as a model system, commenced with reducing the amount of palladium catalyst from 45 mol% to a more typically catalytic quantity of 10 mol%; this gave a correspondingly low yield. Changing the ligand from triphenylphosphine to tri(*o*-tolyl)phosphine had no obvious effect, and neither did the addition of either 1,4-benzoquinone or palladium on carbon as a co-oxidant. However, when the reaction was performed at a three-fold lower concentration (again using 10 mol% catalyst), the yield increased to 68% after purification. At a five-fold lower concentration (approximately 0.01 M), the yield further increased to 74%, and this was conserved when the catalyst loading was reduced to 5 mol%, with a 73% yield obtained. These results thus suggested that this particular intramolecular reaction was especially sensitive to concentration effects. Variation of the ratio of aromatised **19** and non-aromatised products **20** was possibly due to variations in the quality of the nitrogen atmosphere or the palladium source used.

**Intramolecular Heck reaction using the methoxy-substituted bromide 16**

In the specific case of the methoxy-substituted compound, upon subjection to the optimised intramolecular Heck reaction conditions, purified starting material **16** was predominately isolated initially; this suggested that the palladium catalyst underwent faster oxidative addition with the simple benzoquinone **9** than with the alkylated compound **16**. After subjecting this recovered material to the Heck reaction conditions for a second time, the desired products **25-26** were successfully produced, although also with a low mass return.

**NMR spectra of synthesised compounds**

**

**

**

**

**

**

**

**

**

**

**

**

**



**

**

**

**

**

**

**

**

**

**

**

**

**

**

**

**

**

**

**

**

**

**

**

**

**

**

**

**

**

**

**

**

**

**

**

**

**

**

**

























































































**References**

1. Inman M, Moody CJ. Synthesis of indolequinones from bromoquinones and enamines mediated by Cu(OAc)2.H2O. J Org Chem. 2010;75: 6023–6. doi:10.1021/jo101071c

2. Jiao M, Ding C, Zhang A. Facile construction of 3-hydroxyphenanthrene-1,4-diones: Key intermediates to tanshinone I and its A-ring-modified analogue. Tetrahedron. 2014;70: 2976–2981. doi:10.1016/j.tet.2014.03.019

3. Wu N, Ma W-C, Mao S-J, Wu Y, Jin H. Total Synthesis of Tanshinone I. J Nat Prod. 2017;80: 1697–1700. doi:10.1021/acs.jnatprod.7b00238

4. Wu T-S, Jong T-T, Tien H-J, Kuoh C-S, Furukawa H, Lee K-H. Annoquinone-A, an antimicrobial and cytotoxic principle from Annona montana. Phytochemistry. 1987;26: 1623–1625. doi:10.1016/S0031-9422(00)82257-8

5. Krohn K, Loock U, Paavilainen K, Hausen BM, Schmalle HW, Kiesele H. Synthesis and electrochemistry of annoquinone-A, cypripedin methyl ether, denbinobin and related 1,4-phenanthrenequinones. Arkivoc. 2001; 88–130.

6. Fieser LF. Some derivatives of 3,4-phenanthrenequinone. J Am Chem Soc. 1929;51: 940–952. doi:10.1021/ja01378a046

7. Cowden CJ. Use of N-Protected Amino Acids in the Minisci Radical Alkylation. Org Lett. 2003;5: 4497–4499. doi:10.1021/ol035814+

8. Behrman, E. J.; Edwards JO. The thermal decomposition of peroxodisulfate ions. Rev Inorg Chem. 1980;2: 179–206.
